# Comparative Analysis of Species-Specific Ligand Recognition in Toll-Like Receptor 8 Signaling: A Hypothesis

**DOI:** 10.1371/journal.pone.0025118

**Published:** 2011-09-20

**Authors:** Rajiv Gandhi Govindaraj, Balachandran Manavalan, Shaherin Basith, Sangdun Choi

**Affiliations:** Department of Molecular Science and Technology, Ajou University, Suwon, Korea; Centers for Disease Control and Prevention, United States of America

## Abstract

Toll-like receptors (TLRs) play a central role in the innate immune response by recognizing conserved structural patterns in a variety of microbes. TLRs are classified into six families, of which TLR7 family members include TLR7, 8, and 9, which are localized to endolysosomal compartments recognizing viral infection in the form of foreign nucleic acids. In our current study, we focused on TLR8, which has been shown to recognize different types of ligands such as viral or bacterial ssRNA as well as small synthetic molecules. The primary sequences of rodent and non-rodent TLR8s are similar, but the antiviral compound (R848) that activates the TLR8 pathway is species-specific. Moreover, the factors underlying the receptor's species-specificity remain unknown. To this end, comparative homology modeling, molecular dynamics simulations refinement, automated docking and computational mutagenesis studies were employed to probe the intermolecular interactions between this anti-viral compound and TLR8. Furthermore, comparative analyses of modeled TLR8 (rodent and non-rodent) structures have shown that the variation mainly occurs at LRR14-15 (undefined region); hence, we hypothesized that this variation may be the primary reason for the exhibited species-specificity. Our hypothesis was further bolstered by our docking studies, which clearly showed that this undefined region was in close proximity to the ligand-binding site and thus may play a key role in ligand recognition. In addition, the interface between the ligand and TLR8s varied depending upon the amino acid charges, free energy of binding, and interaction surface. Therefore, our current work provides a hypothesis for previous *in vivo* studies in the context of TLR signaling.

## Introduction

Toll-like receptors (TLRs) are pattern-recognition receptors that trigger innate immune responses and prime antigen-specific adaptive immunity [1], [2], [3]. All TLRs have a common domain organization, an extracellular ligand recognition domain consisting of leucine-rich repeats (LRRs), a single transmembrane domain, and an intracellular Toll/interleukin (IL)-1 receptor (TIR) domain [4]. The extracellular domain (ECD) contains repeated LRR modules and is responsible for the recognition of structurally diverse microbial molecules. The basic LRR module is comprised of 24 amino acids that form a β-strand and α-helix joined by a loop, and it is present in several prokaryotic and eukaryotic receptors [5]. Once the TLR ECD (via binding of ligand) is activated, TIR domains dimerize in the cytoplasm, thereby providing a specific scaffold that is required for the binding of downstream adaptor molecules to activate signaling pathways [6]. To date, 10 and 12 functional TLRs have been identified in humans and mice, respectively. TLR1-9 is conserved in both species. However, mouse TLR10 is not functional due to retrovirus insertion, and TLR11-13 have been lost from the human genome [1], [7], [8]. Based on their primary sequences, TLRs can be further divided into several subfamilies, each of which recognizes related PAMPs: the subfamily of TLR2 (TLR2, 1, 6, and 10) is crucial for the recognition of lipoprotein or lipopeptides. The subfamily of TLR4 and 5 recognizes lipopolysaccharides and flagellin, respectively. Viral dsRNA are recognized by the TLR3 subfamily, whereas nucleic acid PAMPs are recognized by the TLR7 subfamily (TLR7, 8 and 9) [9]. Currently, five crystallographic structures of TLR ECDs and their ligand complexes have been reported [10], [11], [12], [13], [14], [15], [16]. Of those, four were found to be complexed with agonistic ligands, whereas the remaining one was complexed with a co-receptor and an antagonistic ligand. These structures provide evidence about how this pattern recognition receptor recognizes “patterns” present in the ligands. Using X-ray crystallographic studies, only a limited number of known TLRs ectodomain interactions with ligands have been observed. Indeed, identification of all ligand interactions of each TLR member using crystallography has been very difficult. Hence, we must rely on molecular modeling and docking studies to gain further insights into these interactions.

Species-specific ligand recognition in TLR biology is an emerging research area for the discovery of novel antagonists and agonists for clinical use, which might lead to the development of new vaccine adjuvants [17]. Previous studies have reported species-specific ligand recognition by TLRs, including: (i) heterodimer complexes of bTLR2/1 and chicken TLR2 type 2/TLR16, which stimulate the transcription factor NF-κB in response to both the tri-acylated lipopeptide Pam3CSK4 and the di-acylated lipopeptide FSL-1 [18], [19], (ii) *Rhodobacter sphaeroides* as an agonist of TLR4 signaling in horses and hamsters and as an antagonist in humans and mice [20], [21], and (iii) bovine and equine TLR4 fails bind with murine TLR4 ligand, taxol [22]. Furthermore, comparative analyses revealed that the function of TLR5 is different in chickens, humans, and mice, indicating species-specificity towards bacterial flagellins [23]. Similarly, non-rodent TLR8s are activated by ssRNA and small synthetic ligands, whereas rodent TLR8s fail to be activated by non-rodent ligands [24], [25]. Such species-specific ligand recognition by TLRs is not often studied, leaving several questions that need to be addressed.

Biochemical studies have shown that TLR8 recognizes ssRNA derived from viruses as well as synthetic small molecules in relation to nucleic acids such as imidazoquinolines and immunostimulatory guanosine nucleotides. However, the structural detail of the ligand-receptor interaction remains unknown. Elucidation of such ligand-binding mechanisms is a necessary step for future studies in order to produce more selective and potent drugs for new potential targets. Moreover, several experimental works have demonstrated that non-rodent (human (h), bovine (b), and porcine (p)) TLR8 signaling is activated by synthetic ligands such as imiquimod (R837), resiquimod (R848), and some guanine nucleotide analogs [26], [27], [28], [29], [30]. However, rodent (mouse (m) and rat (r)) TLR8s, whose primary sequences and structures are identical to non-rodent TLR8s, are not activated by non-rodent ligands. The molecular basis for these binding specificities and affinities are not yet well determined. Therefore, molecular modeling studies are needed to investigate the above phenomenon.

Due to the unavailability of the TLR8 crystal structure, it is very difficult to understand TLR8 receptor-mediated activation. Comparative homology modeling is cited as the most reliable computer-based technique for deciphering the 3D structure of a protein in the absence of its crystal structure. We used this same comparative homology modeling technique to construct the non-rodent and rodent structures of TLR8s based on the known crystal structure of human/mouse TLR3s and polygalacturonase-inhibiting protein [13], [31]. The models were refined by molecular dynamics simulations (MDS), and these refined models were subjected to subsequent molecular docking calculations. Potential TLR8 dimers obtained from the molecular docking calculations were subsequently used for protein-ligand docking to identify the potent binding sites of the antiviral compound R848. Docking studies identified that the ligand-binding site was located near the undefined region, which along with diverse binding affinity might play a key role in TLR8 species-specificity.

## Materials and Methods

### Sequence alignments and comparative homology modeling of TLR8s

TLR8 protein sequences of h, m, r, b, and p were obtained from the NCBI protein database (Accession numbers: GI: 8575527, GI: 14517353, GI: 124245108, GI: 76677887, and GI: 47523453, respectively). The h, m, r, b, and p LRR blocks were annotated using TollML. TollML is a specialized database that organizes the structural motifs of TLRs, derived from the NCBI database [32]. The crystal structures of hTLR3 (PDB ID: 1ZIW) and mTLR3 (PDB ID: 3CIG) were used as common templates to build all of the LRRs of TLR8 species, except the LRR14-15 region. The LRR14-15 region was built using polygalacturonase-inhibiting protein (PDB ID: 1OGQ) as a template. The target-template alignments were carefully checked to avoid deletions or insertions in each block of LRRs and then manually adjusted based upon the sequence alignment obtained from each LRR identified by TollML. Three-dimensional (3D) coordinates of the template proteins were assigned to the target sequences according to the above sequence alignment and then combined to generate multiple alignments. The aligned sequences were then taken for model construction using MODELLER 9v7 [33]. The MODELLER-generated models optimally satisfied spatial restraints, which included homology-derived restraints on the distances, dihedral angels, and stereochemical restraints. Modloop was used to rebuild the model loop regions [34]. The resulting models were subjected to molecular dynamics simulations (MDS) using AMBER99 force field [35] implemented in YASARA dynamics [36].

### Molecular dynamics simulations for TLR8 model

MDS were performed using AMBER99 force field implemented in YASARA. The simulations were performed individually for all five TLR8 species using similar protocols. A simulation cell was constructed around the TLR8 model with a 7.9 Å real space cut-off for the Lennard-Jones forces and the direct space portion of electrostatic forces, which were calculated using the Particle Mesh Ewald method. The pKa values of the ionizable groups were predicted and assigned protonation states based on pH 7.2. The cell was then filled with water, and the AMBER99 electrostatic potential was evaluated for all water molecules; the one with the lowest or highest potential was turned into sodium or chloride counter ion until the cell was neutral. A short steepest descent minimization was done to remove severe bumps, followed by simulated annealing minimizations at 298K, and the velocities were scaled down every ten steps out of 500 steps over a total time period of 5 ps to a final temperature of 0K. We then ran MDS with AMBER99 force field at 298K and 0.9% NaCl in the simulation cell for 1000 ps. The final snapshot was selected based on the lowest potential energy from the 1 ns trajectory, subjected to energy minimization, and subsequently used for molecular docking studies. The refined models were validated using PROCHECK [37], Verify3D plot [38], ERRAT [39], and ProSA z-score [40].

### Protein-protein docking

The refined model was then used for restrained pairwise protein-protein docking to predict the homodimer of TLR8. We used GRAMM-X [41] and Cluspro 2.0 [42], [43], which are widely accepted rigid-body protein-protein docking programs, to predict and access the interactions between the homodimer complexes. Docking sampling was carried out by employing a 128×128×128 point grid with a spacing of 1.2 Å. Both programs produced 10 models, which were ranked as the most probable prediction candidates according to the geometry, hydrophobicity, and electrostatic complementarity of the molecular surfaces, with scoring function used by the programs. To select a model out from the top ten scoring docked complexes yielded by both programs, we applied several criteria, which are mentioned in the results and discussion sections. Energy minimization was performed for the final structures using YASARA package.

### Protein-ligand docking

Docking calculations were carried out using DockingServer [44]. The MMFF94 force field was used for energy minimization of the ligand molecule resiquimod (R848; 4-amino-2-(ethoxymethyl)-a, a-dimethyl- 1H-imidazo [4, 5-c] quinoline-1-ethanol) using DockingServer. Gasteiger partial charges were added to the ligand atoms. Non-polar hydrogen atoms were merged and rotatable bonds were defined. Docking calculations were carried out on the TLR8/TLR8 protein model. Essential hydrogen atoms, Kollman united atom type charges, and solvation parameters were added with the aid of AutoDock tools [45]. Affinity (grid) maps of 27×27×27 Å grid points and 0.375 Å spacing were generated using the Autogrid program. AutoDock parameter set- and distance-dependent dielectric functions were used in the calculation of the van der Waals and electrostatic terms, respectively. Docking simulations were performed using the Lamarckian genetic algorithm (LGA) and the Solis & Wets local search method. Initial position, orientation, and torsions of the ligand molecules were set randomly. Each docking experiment was derived from 100 different runs that were set to terminate after a maximum of 2500000 energy evaluations. The population size was set to 150. During the search, a translational step of 0.2 Å and quaternion and torsion steps of 5 were applied. The molecular graphical representations were prepared using the chimera program. The PyMOL program combined with APBS (http://apbs.sourceforge.net) tools was used to calculate the electrostatic potential at the molecular surface points.

### Computational mutagenesis analysis

The changes in the Gibbs free energy induced by mutation at the undefined region and ligand interaction region of TLR8s were calculated by FoldX [46]. The structures of the five species of TLR8 were minimized using the “RepairPDB” command via YASARA-FoldX plugin to identify the residues that had bad torsion angles, van der Waal's clashes or total energies belonging to the complex interface. Subsequently, individual mutations were built using “BuildModel” command and the ΔΔG values were extracted from the FoldX output files.

## Results and Discussion

### Sequence analysis and comparative model of TLR8

**Figure 1:**
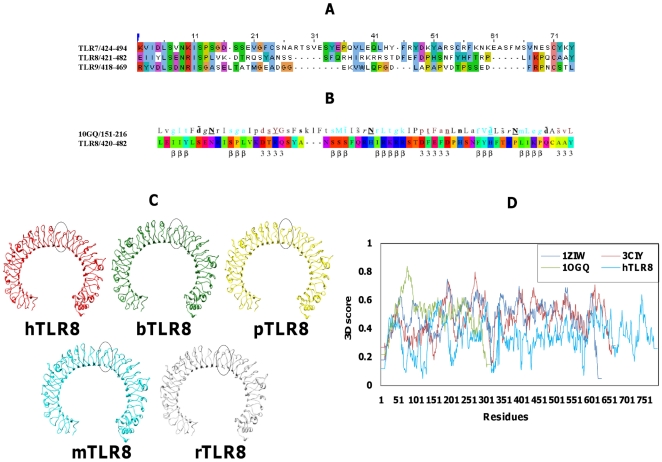
Structure and sequence based alignments. (**A**) Sequence alignment of a region in the TLR7 family members of TLR7, 8, and 9 ECDs centered on LRR14-15. (**B**) LRR14-15 template was identified by using FUGUE server, and this alignment was used to model the LRR14-15 of TLR8s. The sequence indications are specified in Figure 3. (**C**) Ribbon representations of comparative homology modeled structures of rodent (mouse and rat) and non-rodent (human, bovine, and porcine). TLR8s are shown in cyan, brown, red, green, and yellow, respectively. The undefined regions are circled in the TLR8 species. (**D**) Verify 3D analysis of hTLR8 and template structures. Comparison of the calculated averaged 3D profiles of the TLR8 (cyan line) and template structures 1ZIW (blue line), 3CIY (red line), and 1OGQ (green line). The y-axis represents the average 3D-1D score, and x-axis shows residue numbers.

The crystal structures of hTLR3 (1ZIW) and mTLR3 (3CIY) were the two structurally homologous proteins showing higher sequence similarity to the individual target LRRs. Hence, we chose these structures as common templates for constructing the (h, m, r, b, and p) TLR8 model [12], [13], [47]. LRR proteins and domains share a common structure that makes them well suited for mediating protein-protein interactions. Each LRR consists of typically 20–30 amino acids, which include the consensus sequence motif LxxLxLxxNxL (x being any amino acid). These conserved L and N residues can be replaced by other hydrophobic residues. Residues which denote N in the consensus motif play a significant role in forming continuous hydrogen bonds with the backbone carbonyl group of neighboring strands throughout the entire protein, and the resulting structure is referred to as an asparagine ladder. Similar to leucine, the conserved asparagines can be replaced by other residues such as cysteine, threonine, or serine, which are also capable of hydrogen bond formation [5], [48]. As stated by Bell et al. [48], the LRR consensus motifs were found to be conserved in all TLR8 species. The template structure (TLR3), which was composed of 25 LRRs, aligned well with the individual LRRs of hTLR8, except at the LRR14-15 (65 amino acids) region. The region located at LRR14-15, called the “undefined region” [48], [49], [50], exhibited very low sequence similarity between TLR7 family members (Figure 1A). Since a portion of the undefined regions did not align well with the template, we carried out secondary structure prediction to identify structural elements located in this region. The secondary structure prediction revealed that the undefined region was also a prominent feature of the LRRs, consisting of a short β-sheet whose length was 3–5 amino acid residues along with 3_10_ helices (Figure 1B).

Moreover, to build the 3D structure of the unidentified region, a search was conducted to identify the possible template for the undefined region by the fold recognition FUGUE server [51]. We opted for this approach since we could not obtain any relevant structures or domain through Pfam, SMART CDD, and BlastP searches against PDB. The fold recognition server revealed that the template of the undefined region belonged to the LRR family (polygalacturonase-inhibiting protein, 1OGQ) [31]. The target of each LRR sequence aligned with appropriate LRR pairs from the template structures listed in Figure S1 A. Since the accuracy of the model primarily depends on high sequence identity between the target-template alignments, we used manual methods to align the undefined region prior to modeling. The total average target-template identities and similarities of TLR8s were 26.1% and 44.48, respectively (Table 1). We obtained similar target-template similarities and identities among the five different species of TLR8. The optimized sequences were used to construct comparative models by MODELLER 9v4. Our predicted structure of the undefined region agreed well with our secondary structure prediction results. A series of 20 models were built independently, and no difference in the number of secondary structural elements and no significant main chain root mean square deviations (RMSD) were identified. However, the final model was selected based on stereochemical and energetic evaluations. Structural superposition between the modeled TLR8s (h, m, r, b, and p) and the template crystallographic structure of hTLR3 is shown in Figure S1 B. The RMSD values were 1.31 Å (human), 1.25 Å (mouse), 1.24 Å (rat), 1.26 Å (bovine), and 1.24 Å (porcine). Major variations were observed in the undefined regions of the TLR8s when compared to the structure of TLR3. This was mainly due to the presence of irregular LRR domains in the LRR14-15 regions of the TLR8s. Besides, there were minor secondary structural differences observed in the LRR6, 8 and C-terminal regions, whereas the remaining regions aligned well. Notably, these variations occurred away from the ligand-binding regions of the TLR8s. The final model for each species was subjected to molecular dynamics for energy minimization and further optimization.

**Table 1:** Sequence similarities (%) of target-template LRR pairs.

LRRs	hTLR8	LRRs	hTLR8
NT	40	14	35.3
1	45.8	15	41.70
2	32.6	16	52
3	40	17	21.9
4	45.8	18	57.70
5	32.3	19	53.8
6	38.5	20	40.60
7	53.8	21	44
8	23.9	22	33.3
9	46.2	23	54.2
10	55.2	24	60
11	35.3	25	42.30
12	30.8	CT	53.30
13	41.7	Avg	44.48

Note: 1–25 indicate tandem LRRs, NT and CT indicate N-terminal and C-terminal LRRs, respectively. Avg refers to average values.

### TLR8 models refinement by MDS

The constructed models of (h, m, r, b, and p) the TLR8s were subjected to MDS in order to refine the structures for further study. Structural refinement was performed using the explicit solvent method in YASARA program, which automatically ran the simulation for 1 ns to refine each structure; 500 ps was considered to be the equilibration phase while the remaining 500 ps was considered to be production phase (One snap shot as stored every 25 ps and a total of 20 trajectory files were saved). Our MDS trajectory-based analysis showed that the potential energy of the model gradually decreased from −1372500 kJ/mol to −1276900 kJ/mol, which indicates that the model was energetically stable during MDS. An ensemble of 20 structures was obtained using this method, and the representative structure from this ensemble was chosen based on lowest potential energy. Finally, the representative structure was superimposed with ensembles and identified no major structural change in backbone (Figure S2). However, a few side chain clashes were observed in the remaining ensembles.

The representative structure was subjected to energy minimization followed by stereochemical quality evaluation (Figure 1C). The quality of the protein geometry was checked by employing ERRAT Protein Verification, PROCHECK, Verify 3D, and ProSA z-score. The Ramachandran plot calculations were computed with the PROCHECK program, which checks the detailed residue-by-residue stereochemical quality of a protein structure. The ψ and φ distributions of the Ramachandran plot of the non-glycine, non-proline residues are summarized in Table 2. Altogether, >90% of the residues in the homology models were in favored and allowed regions. This analysis shows that the template and homology models possess similar Ramachandran plots with a relatively low percentage of residues having general torsion angles. The ERRAT program is a so-called “Overall quality factor” that works by analyzing the statistics of non-bonded interactions between different atom types, with higher scores depicting higher quality structures. In the current case, the ERRAT score for all of the models was >70%, well within the range of a high quality model (Table 2). Compared to the template, the ERRAT scores for the models suggest that the backbone conformation and non-bonded interactions of the homology models were all within a normal range. ProSA calculated the interaction energy per residue. In this analysis, the interaction energy of each residue with the remainder of the protein is computed in order to determine whether or not it fulfills certain energy criteria. In the TLR8s homology model, the resulting z-score was around −6, similar to that of the template, further confirming that the energy profile of the models is consistent with a reliable conformation based on similarity with that of the templates (Table 2). Verify 3D analysis of the models found no conformational errors, and there were no values less than 0.09, further indicating that all of the residues were located in favorable structural environments (Figure 1D). Similar results of PROCHECK and Verify 3D were observed for the remaining TLR8 species. The Verify 3D, PROCHECK, ERRAT, and ProSA z-score results together show that the representative structure was satisfactory and can thus be considered as a reliable source for further analyses.

**Table 2:** Model validations and comparison with templates.

	PROCHECK							ERRAT	ProSA
	Ramachandran plot statistic (%)				Goodness factor				
	Core	Allowed	General	Disallowed	Dihedrals	Covalent	Overall	Score	z-score
hTLR8	78	20.9	1.1	0	0.01	0.31	0.06	80.244	−6.43
mTLR8	78.5	20.1	1.4	0	−0.34	0.47	0.07	76.407	−6.67
rTLR8	78.3	20	1.7	0	−0.41	0.54	0.03	74.073	−6.85
bTLR8	79	19.8	1.3	0	−0.38	−0.24	0.18	69.06	−6.72
pTLR8	79.4	19	1.5	0	−0.36	−0.51	0.04	69.43	−6.47
10GQ	77.4	22.3	0.4	0	−0.18	−0.29	0.2	84.918	−8.64
1ZIW	75.5	24.2	0.3	0	−0.36	0.35	−0.08	68.908	−6.89
3CIY	64.1	34.6	1.2	0.2	−0.24	0.47	0.04	80.682	−7.89

Note: Ramachandran plot qualities show the percentage (%) of residues belonging to the core, allowed, generally allowed, and disallowed region of the plot: goodness factors show the quality of covalent and overall bond/angle distances: these scores should be above −0.5 for a reliable model. ERRAT and ProSA score indicates the calculated overall quality score for protein structures.

### Dimerization of TLR8/TLR8 complex and MDS

**Figure 2:**
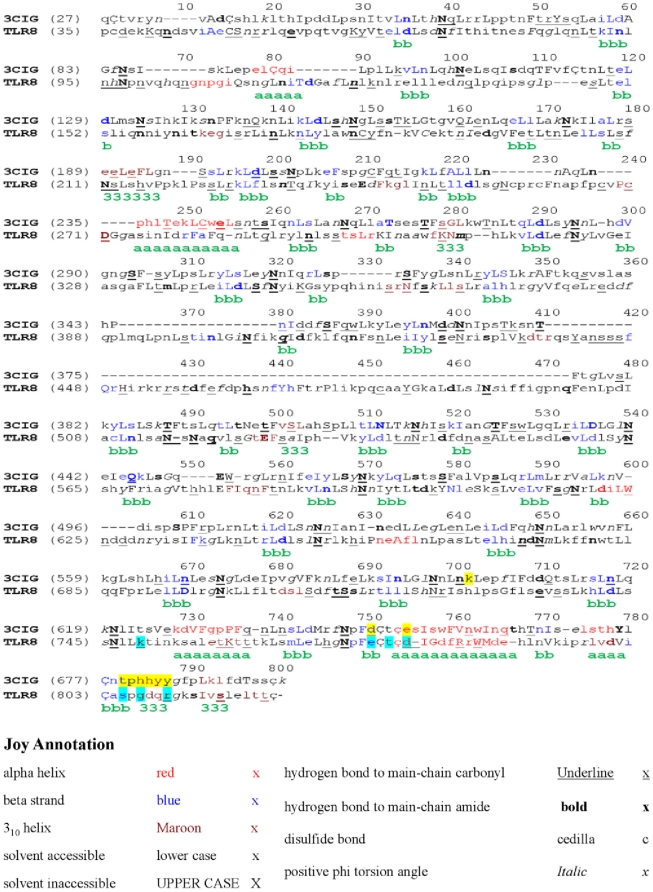
The structure-based sequence alignment between TLR8 and TLR3. The local structural environment of each residue is displayed using JOY annotation. The helix regions (shown in red) tend to be less in number than the loop regions (shown in black). The TLR3 and TLR8 residues involved in C-terminal interactions are highlighted in yellow and cyan, respectively.

Recently solved crystal structures of TLR1/2, TLR2/6, TLR4-MD-2-LPS, and TLR3 have shown that TLR exists as a monomer in solution and that dimerization takes place only upon ligand binding [3], [10], [11], [12], [52], [53]. The ligand-induced dimerization of the TLR-TLR ectodomains causes the juxtamembrane sequences in the C-terminal ectodomains to come into close proximity across the transmembrane, resulting in reorientation or homodimerization between the TLR TIR domains. The homodimeric TIR receptor provides a specific molecular surface for the recruitment of TIR domains containing signaling adaptor proteins [47]. Conversely, TLR7 subfamily members (TLR9 and 8) exist as a preformed dimer prior to ligand recognition. The binding of ligand induces conformational changes in the ectodomain, leading to dimerization of the TIR domain and initiation of downstream signaling [54]. Since the formation of the TLR8/TLR8 complex is necessary for ligand binding, we modeled the TLR8 homodimer through protein-protein docking methods. TLR3 is closely related to the TLR7/8/9 family because of its intracellular localization and nucleic acid ligand. Therefore, we used the recently published crystal structure of the TLR3/TLR3-dsRNA complex as a guide to predict the essential interacting dimerization residues in TLR8 and used those residues as a constraint during protein-protein docking study. Structure-based sequence alignment of TLR3 and 8 are shown in Figure 2. The residues involved in TLR3 dimerization are located at the C-terminus (shaded in yellow), and the corresponding residues in TLR8 are shaded in cyan. In order to verify the prediction confidence of the TLR homodimer interaction, protein-protein docking methods, GRAMM-X and Cluspro 2.0, were used. To confirm the accuracy of these methods, we performed unrestrained rigid-body docking for TLR3 dimer as a test case, for which the dimer crystal structure is known. The native TLR3 homodimer structure was present in the top 10 solution of both GRAMM-X and Cluspro 2.0 and was ranked ninth and sixth by GRAMM-X and Cluspro 2.0, respectively. This test highlights the feasibility and reliability of GRAMM-X and Cluspro 2.0 in TLR-TLR docking; hence, we used these methods in our subsequent docking calculations.

**Figure 3:**
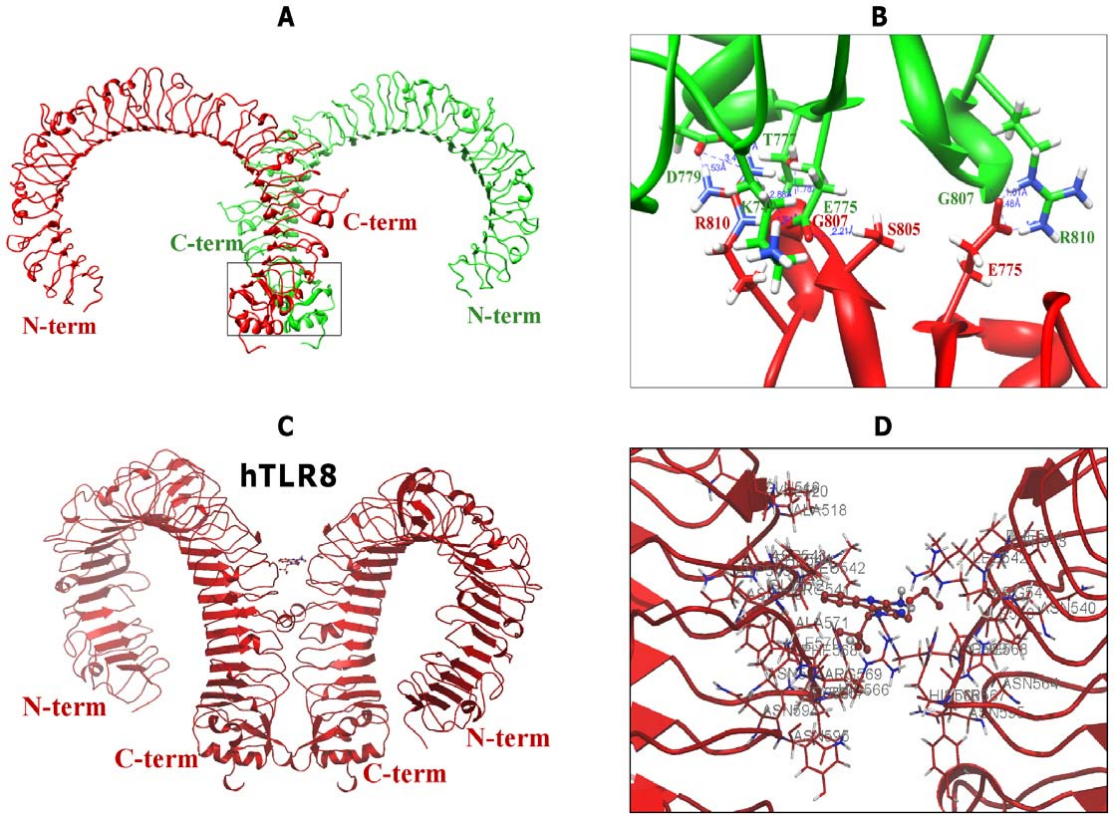
Docked poses of hTLR8 dimerization and ligand interaction. (**A**) A homodimer observed in the docking methods is shown with one monomer in green (B chain) and the other in red (A chain). (**B**) Close-up view of the C-terminal domain-interacting residues. Hydrogen bonds are represented by blue colored dashed lines. Key residues taking part in hydrophobic interactions as well as salt bridges are shown. (**C**) The side view docking poses for R848 in human TLR8/TLR8. (**D**) Possible mode of R848 and its interaction with human TLR8/TLR8. The protein dimer backbone is represented as a red ribbon; R848 is represented in stick format and colored in red, with the interacting residues colored in red and labeled.

We subsequently used unrestrained docking to obtain the TLR8 homodimer using the docking programs (GRAMM-X and Cluspro 2.0). Both docking programs produced top 100 solutions of homodimer complexes for each TLR8 species, but none of the complexes correlated with the available experimental evidence. Therefore, we used restrained docking methods (GRAMM-X and Cluspro 2.0) to predict the near native-like homodimer structures of TLR8s. Each program returned 30 complexes, of which most possessed an ‘m’ shaped dimer. We chose our desired complexes based upon the following criteria: (i) Shared models produced by two programs (ranked third and seventh by GRAMM-X and Cluspro, respectively) (ii) From these shared models, we chose the final docked complex based upon the high buried surface area. We superimposed similar ‘m’ shaped complexes, which were produced by both docking programs, and the selected final complexes are shown in red (Figure S3). The protein-protein interfaces of the final docked complexes were further analyzed using the PISA server (http://www.ebi.ac.uk/pdbe/prot_int/pistart.html). The final docked complexes for all of the species were obtained using a similar protocol, and their ranks in the docking program are listed in Table S1. As expected, the orientation of the TLR8/TLR8 complex resembled the crystal dimer structure of TLR3. The dimerization mainly occurred in the C-terminal region (Figure 3A & Table 3), whose residues were highly conserved among TLR8 species (Table S2).

**Table 3:** hTLR8-hTLR8 dimer interface.

Molecule A	Chain	Distance [Å]	Molecule B	Chain
R810	A	2.01	E775	B
G807	A	1.89	E775	B
R810	A	2.88	E775	B
E775	A	1.73	S805	B
T777	A	1.92	G807	B
E775	A	1.93	R810	B
D779	A	2.89	R810	B
E775	A	2.91	R810	B
D779	A	3.09	R810	B
K749	A	3.38	R810	B

Note: Residues involved in C-terminal protein-protein interactions. Cutoff distance is 3.5 Å. Molecule A and B chains represent two monomer chains of hTLR8.

The final TLR8 dimer structure orientation was similar to all experimentally solved TLRs with an “m” shaped dimer architecture. However, the distance between the TLR3 and TLR8 dimer surfaces was reasonably diverse (∼90 Å for TLR8). Conversely, the TLR3 homodimer was separated by ∼120 Å. Therefore, the dimer structure possessed a wide-open surface for binding with 40–50 base pairs of dsRNA. The modeled TLR8 dimer structure had a slight crevice on its surface, which accounts for the disparity observed in the surface curve. As noted, TLR8 mediates anti-viral immunity and anti-tumor responses by recognizing ssRNA viruses and synthetic small molecular weight ligands, respectively. The superimposition of TLR3 with the TLR8 revealed that the cavity formed by the TLR8 monomer surfaces was smaller when compared to the TLR3 dimer structure, and therefore TLR8 was able to interact with both physiological (ssRNA) and synthetic ligands (imidazoquinolines) (Figure S4). The obtained docked complexes of TLR8 dimers from all of the species were reasonable; hence, we tested these complexes for binding with R848 in ligand docking studies.

### Protein-ligand docking studies

The quality of the backbone conformation, residual interactions, residual contacts, and the dynamic stability of the structures were all well within the limits established for reliable structures. This suggests that an adequate dimer model for TLR8s can be obtained to characterize its potential ligand interactions and investigate species-specificity. In order to understand the species-specificity of ligand recognition in TLR8 signaling, to select the best TLR8 dimer model, and to understand its binding behavior in terms of affinity as well as selectivity, we carried out docking with a known synthetic small molecule agonist, R848 (as detailed in the methods section). Recent biochemical studies found that R848 activates TLR8 in macrophages, resulting in the production of cytokines such as IFN-α, IFN-γ, TNF-α, and IL-12. R848 increases cellular immunity when compared to structurally similar R837 [55], [56], [57], [58]. The current docking protocol has been validated using the known crystal structure of TLR3 homodimer with dsRNA by DockingServer [44]. This program produced 100 complexes (based on the scoring function), of which the near native homodimer structure of the TLR3/dsRNA complex ranked third. We therefore concluded that our docking protocol is reliable and can be subsequently used for TLR8 species. In general, the docking program produces poses with same or different orientations within the defined active site. We chose final complexes of each TLR8 species based on criteria such as free energy of binding, interaction surface, binding affinity, pose of the docked ligands, complex geometry, and large interaction energy (Figure S5).

**Figure 4:**
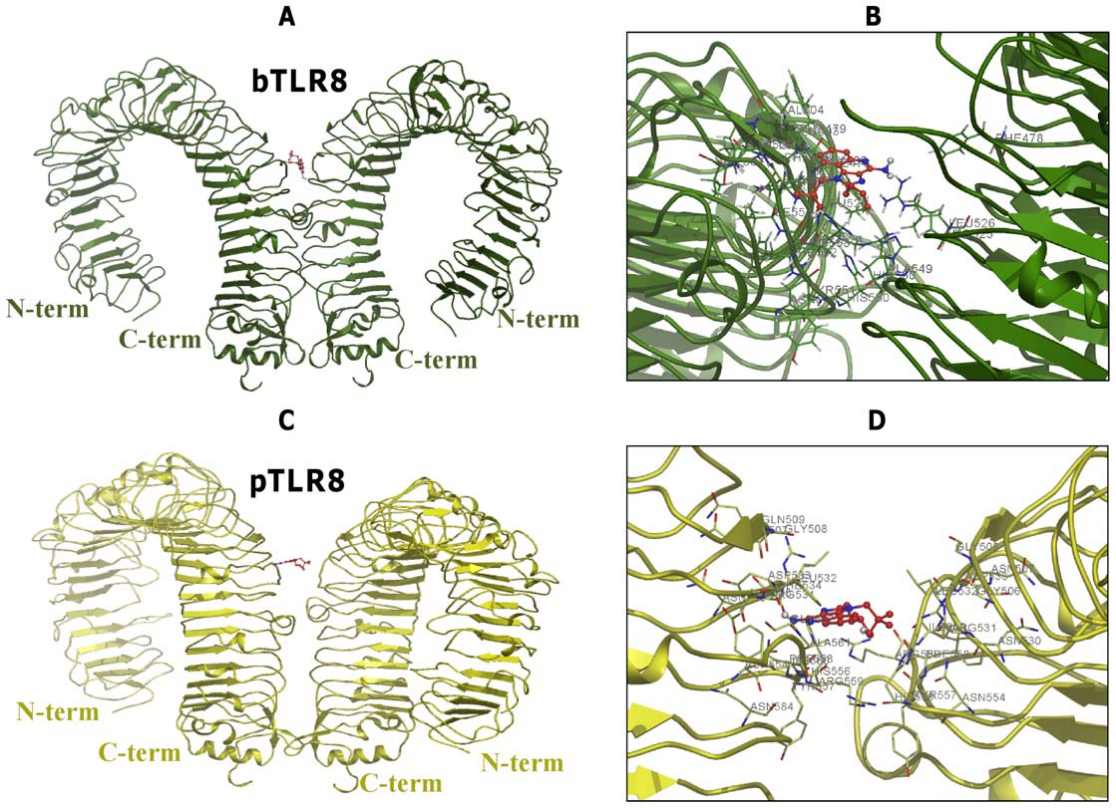
Models of binding of R848 to bovine and porcine homodimer of TLR8s. (**A**) The side view docking poses for R848 in bovine TLR8/TLR8. (**B**) Detailed top view of the R848 docking pose in bTLR8/bTLR8. The protein dimer backbone is shown as a green ribbon; R848 is shown as sticks and colored in red, with the interacting residues colored in green and labeled. (**C**) The side view docking poses for R848 in porcine TLR8s. (**D**) Detailed top view of the R848 docking pose in pTLR8/pTLR8. The protein dimer backbone is shown as a yellow ribbon; R848 is shown as sticks are colored in red, with the interacting residues colored in yellow and labeled.

**Figure 5:**
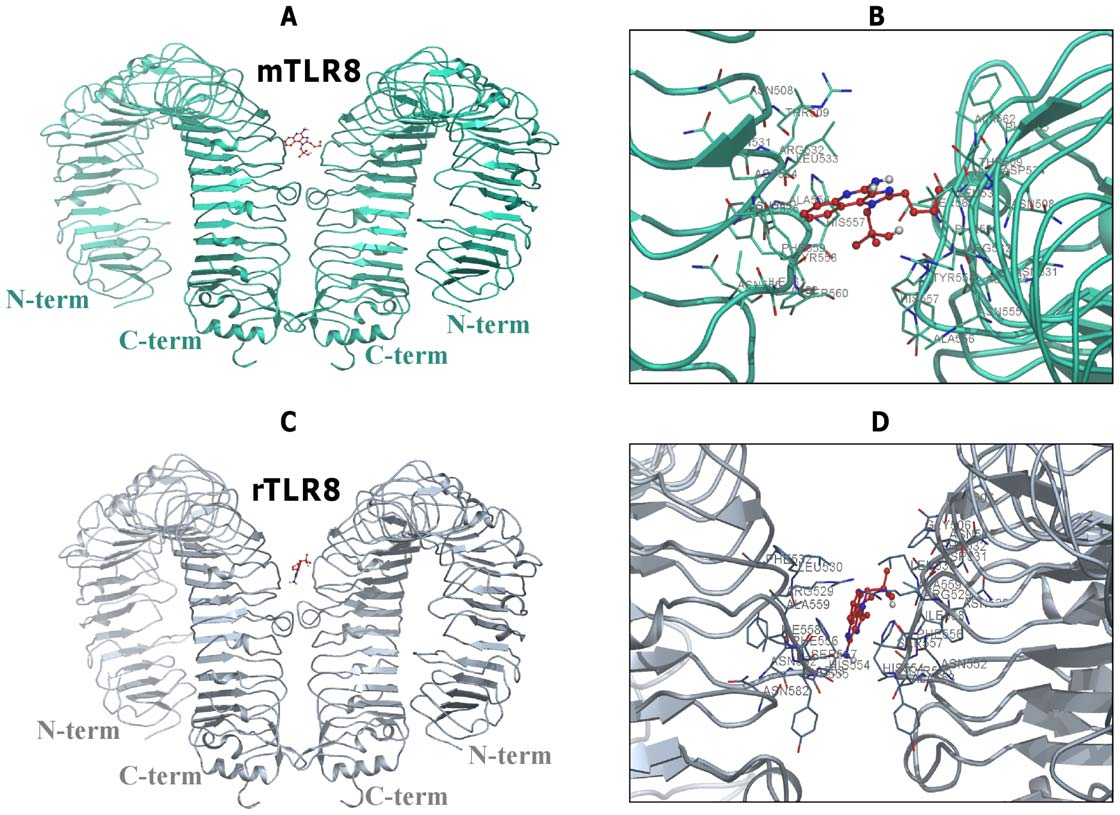
Proposed model of R848 with rodent TLR8s. (**A**) The side view docking poses for R848 in mouse TLR8/TLR8. (**B**) Detailed top view of the R848 docking pose in mTLR8/mTLR8. The protein dimer backbone is shown as a cyan ribbon; R848 is shown as sticks are colored in red, with the interacting residues colored in cyan and labeled. (**C**) The side view docking poses for R848 in rat TLR8s. (**D**) Detailed top view of the R848 docking pose in rTLR8/rTLR8. The protein dimer backbone is shown as a brown ribbon; R848 is shown as sticks are colored in red, with the interacting residues colored in brown and labeled.

Docking studies placed R848 ligand in the TLR8 homodimer active site, whose binding orientations are more or less similar to the crystal structure of TLR3/dsRNA homodimer. It should be noted that in TLR3, both the N-terminal and C-terminal portions are involved in ligand binding [12]. However, in our predicted complexes (TLR8/TLR8-R848), only the C-terminal region was involved in ligand interaction, mainly due to the nature of the ligand molecule (Figures 3, 4, 5). Recent findings on TLR8 revealed that the N and C-terminal regions also participate in ligand recognition when interacting with large-sized ligands such as ssRNA [49]. The H-bond existing between synthetic R848 and TLR8s species is shown in Figures 3, 4, 5. The amino acids that play a pivotal role in R848 recognition in the five species are listed in Tables S3, S4, S5, S6, S7. It has been reported that low pH is a necessary prerequisite for signaling to occur in the TLR7 family. Ruiz et al. proposed that CpG DNA binds to TLR9 in a pH-dependent manner, and it interacts weakly with CpG DNA at physiological pH [59]. In this regard, similar to TLR9, TLR8 mediates signaling in a pH-dependent manner [49]. The binding specificity may depend on the ionization state of the CpG DNA or R848 and ssRNA in the case of TLR8. R848 is a membrane permeable weak base ligand with a number of ionizable groups and a pKa value of 7.2, which binds to the negatively charged TLR8 receptor. Interestingly, our docked model of TLR8/TLR8-R848 demonstrates that the positively charged antiviral drug R848 formed a strong electrostatic interaction with the negatively charged residues of D543 in TLR8 (Figures 3, 4, 5). Moreover, previous mutational and computational analyses found that the amino acids D543, S492, Q519, N539, R541, F544, and H566 of TLR8 are indispensable for interaction with different ligands [24], [60], [61]. Our docked complex revealed that except for S492 and N539, the remaining residues identified in mutational studies participated in various interactions with R848. Therefore, the residues (S492 and N539) that were not involved in the above interactions might possibly interact with ssRNA ligand. Furthermore, our docked complex illustrated that the interaction of TLR8 with its ligand was mainly mediated through hydrophilic and electrostatic interactions.

### Identification of functional residues by computational mutagenesis

The effect of the undefined region residues (438–442) on human TLR8 has been investigated previously [60], and it was found that these mutations led to strong effects on human TLR8 activation. However, multiple sequence alignment indicated that this undefined region is conserved in non-rodent species of TLR8, whereas these residues were absent in rodent species (Figure S6). Moreover, it remains unclear the role of undefined region in non-rodent paralogs and rodent TLR8s activation. To elucidate this problem, computational mutagenesis was carried out by FoldX [46], a well-established method that has been successfully applied to the analysis of protein folding, protein design, protein-protein interactions, protein-DNA binding and evolution in a variety of proteins. The prediction was carried out using YASARA plug-in FoldX procedures that used the “RepairPDB” command to minimize the energy of a protein structure by rearranging the amino acid side chains in order to get a better free energy of the protein before the calculation of FoldX mutational analysis for hTLR8. Mutagenesis was carried out using the BuildModel FoldX command, and each mutation was repeated for five times in each structure. A threshold of 1.6 kcal/mol was considered, as it corresponds to twice the standard deviation calculated with FoldX and values above this threshold were considered to destabilize the protein.

**Figure 6:**
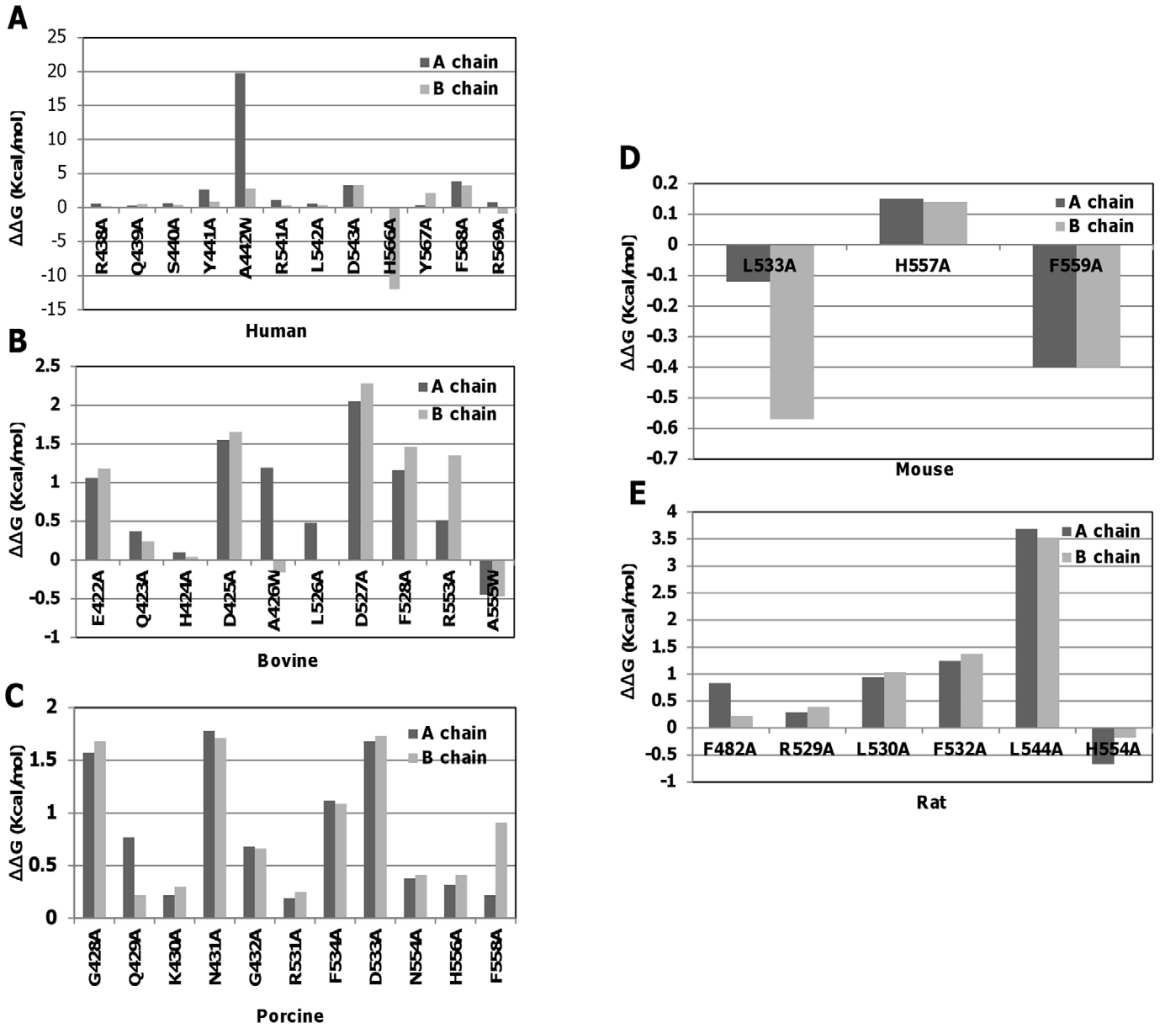
The contribution of each residual activity induced by mutations predicted by FoldX. Plot of calculated ΔΔG values for both undefined and ligand interaction region residues of (**A**) hTLR8, (**B**) bTLR8 and (**C**) pTLR8. Plot of calculated ΔΔG values for only ligand interaction residues of (**D**) mTLR8 and (**E**) rTLR8.

As shown in Figure 6, the mutations were tested for the undefined region residues and ligand interaction residues of TLR8 which were predicted by our docking analysis. Figure 6A clearly indicates that all the mutations tested for hTLR8 had small ΔΔG values around 1 kcal/mol and the large change in stability were found to be caused by the chains of Y441 chain A (2.66 kcal/mol), R541 chain B (1.63 kcal/mol), D543 chain A (3.21 kcal/mol), D543 chain B (3.33 kcal/mol), F568 chain A (3.87 kcal/mol), F568 chain B (3.23 kcal/mol), whereas the replacement of A442 to W significantly affected the overall stability of the protein (19.79 kcal/mol - chain A) (2.8 kcal/mol - chain B). These results suggested that according to the FoldX prediction, the substitution of Ala residues at the undefined and ligand interaction region significantly affects the TLR8 activation. The theoretical determination of FoldX alanine mutational analysis for hTLR8 has been previously validated by experimental data showing high degree accuracy [39]. In this sense, the theoretical value of undefined region residues had larger ΔΔG values that were consistent with the experimental data further highlighting the importance of undefined residues for hTLR8 activation [60]. Hence, we extended our analysis to other species of TLR8. Figure 6B shows that the bTLR8 had small ΔΔG values around 1 kcal/mol and the largest changes in stability were observed for the A and B chains of E422 (1.76 kcal/mol) (1.88 kcal/mol), D425 (1.75 kcal/mol) (1.65 kcal/mol), D527 (2.05 kcal/mol) (2.28 kcal/mol) and F528 (1.66 kcal/mol) (1.96 kcal/mol), respectively. Figure 6C shows that the pTLR8 had small ΔΔG values around 1 kcal/mol and the largest changes in stability were observed for the A and B chains of G428 (1.57 kcal/mol) (1.68 kcal/mol), N431 (1.78 kcal/mol) (1.71 kcal/mol) and D533 (1.68 kcal/mol) (1.73 kcal/mol), respectively. Both these results were consistent with the experimental data further emphasizing the role of undefined region in protein stability [25], [60]. Interestingly, D543 which is suggested by earlier experimental analysis showed that this residue could participate in ligand interaction and had effect on protein stability which correlated well with our FoldX analysis. Furthermore, this D543 corresponding residues in other species that were involved in ligand interaction also had large ΔΔG values which were consistent with previous experimental prediction [24], [25], [60]. Since, the undefined region residues are absent in rodent species, we have focused on the ligand interaction regions. As shown in Figure 6D, all the mutations tested in mTLR8 that participated in ligand interaction had very small ΔΔG cut off value, whereas the rTLR8 (Figure 6E) had a small ΔΔG value around 1 kcal/mol and the largest changes in stability were found to be due to the A and B chains of F532 (1.24 kcal/mol) (1.37 kcal/mol) and L544 (3.69 kcal/mol) (3.53 kcal/mol), respectively. Thus, our results indicate that all mutations had minor effect in rodent TLR8s, whereas it showed a large effect in non-rodent TLR8s stability.

### Species-specific ligand recognition

**Figure 7:**
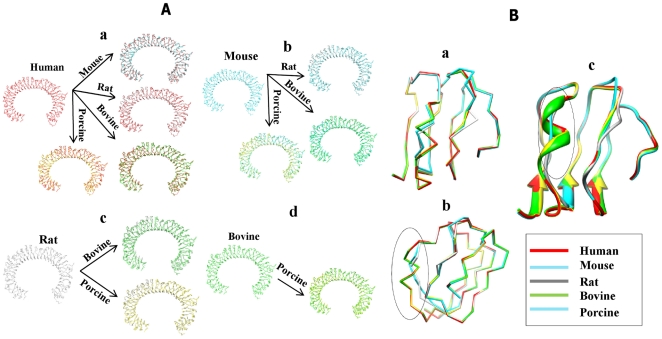
Structural superimposition of the TLR8s used in the analysis. (**A**) The overall topologies of the TLR8s are remarkably similar, with LRRs superimposing relatively well in most cases apart from the undefined region (LRR14-15). (a) The structural comparison of the human TLR8 with mouse, rat, bovine, and porcine, (d) mouse TLR8 with rat, bovine, and porcine, (c) rat TLR8 with bovine and porcine, (d) bovine TLR8 with porcine. The human, mouse, rat, bovine, and porcine TLR8 are colored in red, cyan, gray, green, and yellow, respectively. (**B**) Superimposition of the undefined region (LRR14-15) of hTLR8 with other species shows significant differences. (a) All species undefined regions are shown in alpha-carbon trace, (b) side view of the structural differences in the undefined regions are circled, (c) superimposition of the undefined regions shows non-rodent (mouse and rat) that lacks a coil region are circled.

Perhaps the most interesting aspect of this work is the finding that structural differences among species can lead to variable specificity in ligand recognition. We superimposed the refined structures of the TLR8s from rodent and non-rodent (Figure 7A), whose structural superimposition and sequence identity is shown in Table 4. This analysis revealed that hTLR8 was structurally identical with other models in all of the LRR regions, except the LRR14-15 portion (residues 438–442) (Figure 7A). Moreover, interaction residues that participated in the docked complex of TLR8/TLR8-R848 were conserved among all of the species, except for residues N492 and S569 from rodent (Table 5). Although, high sequence identity was observed between rodent and non-rodent TLR8s, there were some residual variations (RQSYA (residues 438–442)) among these species in their undefined regions (Figure S6). We therefore superimposed this portion from all of the species and found that non-rodent TLR8s possessed a coiled region (residues 438–442) in the undefined region, whereas the rodent TLR8s possessed a loop (Figure 7B). We hypothesized that such structural discrimination in the undefined region between rodent and non-rodent might possibly play a key role in species-specificity. However, these regions did not play any role in ligand interaction. Moreover, in our docked complexes, the ligand-binding cavity was located in LRR15-17 (residues 484–572), which was in close proximity to the undefined regions. It is of note that the surface charges present in the undefined region might possibly be a deciding factor affecting species-specificity. Hence, we carried out electrostatic potential studies for all TLR8 species.

**Table 4:** Structural and sequence comparison among the TLR8 ECD species.

Species	Species	RMSD [Å]	Sequence identity (%)
hTLR8	mTLR8	0.409	67
	rTLR8	0.71	68
	bTLR8	0.734	71
	pTLR8	0.417	70
mTLR8	rTLR8	0.911	86
	bTLR8	0.697	64
	pTLR8	0.445	67
rTLR8	bTLR8	0.894	64
	pTLR8	0.715	67
bTLR8	pTLR8	0.746	74

Note: hTLR8, mTLR8, rTLR8, bTLR8, and pTLR8 indicate human, mouse, rat, bovine, and porcine Toll-like receptor 8, respectively. RMSD value denotes the root mean square deviation value between the Cα atoms of species. % denotes sequence identity in a percentage.

**Table 5:** The ligand-binding region of TLR8 among the species.

Position	hTLR8	mTLR8	rTLR8	bTLR8	pTLR8
492	S	***N***	***N***	S	S
519	Q	Q	Q	Q	Q
539	N	N	N	N	N
541	R	R	R	R	R
542	L	L	L	L	L
543	D	D	D	D	D
544	F	F	F	F	F
566	H	H	H	H	H
567	Y	Y	Y	Y	Y
568	F	F	F	F	F
569	R	***S***	***S***	R	R

Note: The ligand-binding residues not conserved among the species are shown in boldface with italic. hTLR8, mTLR8, rTLR8, bTLR8, and pTLR8 indicate human, mouse, rat, bovine, and porcine Toll-like receptor 8, respectively.

**Figure 8:**
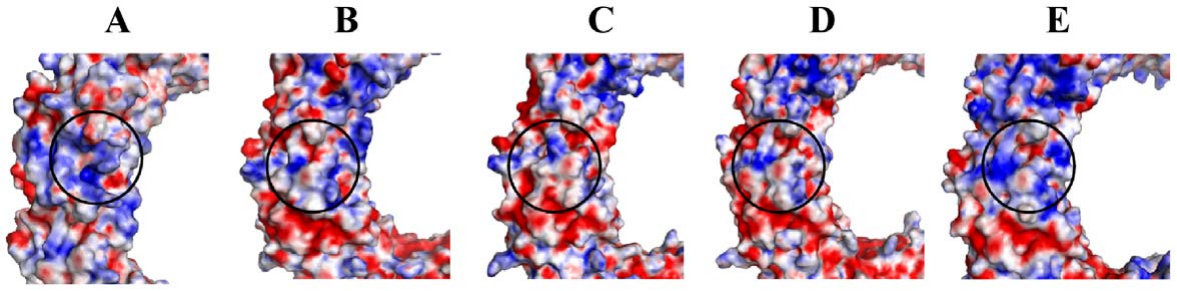
Distinct electrostatic surface potentials of TLR8 from different species. TLR8 LRRs 14-17 species differences in shape and electrostatic surface potential are shown. The concavity-located residues are circled. Electrostatic surface potential represented for the TLR-characteristic structures of human (h), mouse (m), rat (r), bovine (b), and porcine (p) of TLR8 are shown.

Electrostatic potential surface of TLR8s from different species revealed significant differences in the undefined region located near the ligand-binding cavity (Figure 8). Our hypothesis correlated with previous experimental results, in which authors proposed that undefined residues from 438–442 are required for TLR8 activation but are not involved in ligand interaction. Taken together, our data suggest that charge differences between the TLR8s of the rodent and non-rodent species might be the deciding factor governing the pharmacology of R848 through the TLR8 signaling pathway.

The TLR8/TLR8-R848 docked complex revealed that the ligand-interacting residues present in hTLR8 were conserved among the species (Table 5). Previous biochemical studies suggested that D543 is a critical residue in hTLR8 and determines ligand activity. Our docking studies found that D543 was located in the cavity and played an important role in forming an H-bonding network to R848, thereby activating TLR8 ECD. Interestingly, D543 residue of hTLR8, whose corresponding residues in bTLR8 and pTLR8 are D527 and D533, respectively, formed strong H-bonds with ligand. However, those critical corresponding residues (D534 and D531) found in rodent TLR8s did not have any contact with ligand due to their different conformational ligand binding (Table 5 and Tables S2, S3, S4, S5).

The orientations of identified docked complexes (TLR8/TLR8-R848) are similar to that of the TLR3/TLR3-dsRNA complex [12]. However, the binding poses of rodent and non-rodent TLR8s were slightly dissimilar, which we suspect causes variation in the interaction energies for each complex, leading to the inability of rodent TLR8s to activate TLR8 signaling pathways. To this end, we calculated the binding affinity, intramolecular energy, van der Waals potential energy, H-bond of desolvation energy and interaction surface of each pose of the receptor (Table 6). Our analyses revealed that non-rodent species had higher binding affinity than rodent TLR8s. Due to their lower binding affinity, rodent TLR8s were not able to activate the TLR8 signaling pathway, which is consistent with previous reports. Therefore, this report proposes that rodent and non-rodent TLR8s bind with their ligands, although rodent TLR8s have weaker signaling initiators when compared to non-rodent TLR8s [60].

**Table 6:** Results of docking calculations using Gasteiger charges on both the ligand and protein.

Protein-Ligand complexes	Free Energy of Binding cal/mol	vdW+Hbond+desolvation Energy kcal/mol	Total intermolecular. Energy kcal/mol	Interaction Surface
hTLR8/R848	−6.85	−8.34	−8.44	737.624
bTLR8/R848	−5.40	−6.82	−6.80	650.436
pTLR8/R848	−6.69	−8.09	−8.18	732.534
mTLR8/R848	−4.57	−6.67	−6.72	708.909
rTLR8/R848	−4.91	−6.21	−6.28	670.738

Note: hTLR8, mTLR8, rTLR8, bTLR8, and pTLR8 indicate human, mouse, rat, bovine, and porcine Toll-like receptor 8, respectively.

In contrast, rodent TLR8s were believed to be non-functional as it cannot be activated by RNA or small molecular ligands. However, it was recently shown to be activated by ligands when used in combination with polyT oligodeoxynucleotides (ODNs) [62]. Furthermore, Gao et al., found that a series of imidazoquinoline derivatives act as allosteric enhancers of agonist binding at human A3 adenosine receptors [63], which indicates that ODNs and imidazoquinoline molecules may directly bind to the TLR8. Binding of polyT ODN to TLR8 can act as an (allosteric) activator enabling increased binding of small molecule ligands to the active TLR8 binding pocket for increased downstream signaling. This study correlates with our hypothesis that non-rodent ligands can alone bind with rodent TLR8s, however the signaling mechanism is not activated, This finding indicates that non-rodent ligands need to combine with polyT ODNs in order to stimulate the rodent TLR8s. Few reports have suggested that there is a direct interaction between the small molecule ligands and polyT ODNs that might be responsible for the rodent TLR8s to be functional [64]. Hence, polyT ODNs play a key role to activate the rodent TLR8s that is mainly responsible for the differences observed in the species-specificity with both synthetic and physiological ligands. Despite the structural similarities between TLR7 and TLR8, their activation has distinct consequences on the innate immune cells and subsequent production of cytokines [65]. Interestingly, imiquimod activates preferentially TLR7; its agonistic activity at TLR8 appears to be much weaker [66]. It is therefore prudent to study the mechanism of action of these drugs.

Besides the species-specificity between rodent and non-rodent ligand recognition, there has been diverse ligand recognition patterns observed between the non-rodent TLR8s (h, b, and p). hTLR8 can be activated by polyT ODNs, R848 and CL075, but not imiquimod, whereas b and pTLR8 are activated by all human-activating ligands and also by imiquimod [24], [25]. However, imiquimod is able to activate the human TLR8s when in combination with polyT ODNs [66]. Notably, the comparative analysis of non-rodent TLR8s within the undefined region demonstrated low sequence similarity among the non-rodent species, leading to diverse ligand recognition among the non-rodent TLR8s (Figure 7B). Only a few amino acids clustered around the binding site differed between TLR8 from different species, and such variation was necessary for species-specificity. Moreover, our docking study calculations also showed that there were slight differences in ligand-binding affinity between non-rodent TLR8s (Table 6). Although, bTLR8 was able to bind with its ligand similar to other non-rodent (h and p) TLR8s, its binding affinity was lower. This observation is consistent with a previous work in which R848 induced human and sheep TLR8 10-15 fold and bovine and cat TLR8 were induced less than 5-fold [60]. In addition, the phylogenetic analysis of TLR8 species also revealed that non-rodent and rodent species are evolutionarily diverse (Figure S7). Previous reports found that TLR5 mediates species-specificity in flagellin, and ligand recognition is controlled by residues surrounding its binding site. [23]. Our hypothesis also coincides with the above TLR5 studies that found that concavity surrounded regions are responsible for mediating species-specificity in ligand recognition. Taken together, these preliminary comparative studies on TLR8s suggest that general TLR ligand recognition and subsequent signaling could be species-specific.

In our current study, we used computational modeling studies to build a homodimer complex structure of TLR8 with the antiviral drug R848. The potential TLR8/TLR8-R848 complexes were able to explain the species-specificity of TLR8 activation by its ligand, which is in agreement with previous mutagenesis studies. Our comparative studies on TLR8s suggest that insertion mainly takes place in an undefined region shared across species that possesses variable charge and secondary structural elements. This undefined region located near the active site, along with the active site surface residues, might play a key role in species-specificity. Our current models can be utilized as a guide for future experimental and computational studies to draw biological and functional conclusions. The presented modeling approach can be extended to other repetitive TLR protein ectodomains.

## Supporting Information

Figure S1 **Multiple sequence and structural alignments between targate and templates.** (**A**) Three templates, mTLR3 (3CIY), hTLR3 (1ZIW), and P. vulgarism polygalacturonase-inhibiting protein (1OGQ), were used to construct the model of the TLR8s. The number 1–25 indicates the canonical LRRs, and NT and CT indicate N- and C-terminal LRRs, respectively. (**B**) Superimposition between crystal TLR3 (red) and the MD-refined TLR8 models.
(TIF)

Figure S2 **MDS-refined average structures of clusters.** MDS produced 20 refined models of hTLR8 shown in alpha-carbon. Reference structure is cyan; the selected average structure of the cluster is red. Similar refined structures produced by MDS for the remaining TLR8 species were used for further studies.
(TIF)

Figure S3 **Top-ranked docked complex comparison with final selected complexes.** (**A**) The final selected hTLR8 complex superimposed with similar orientation complexes yielded by both docking programs. A chosen hTLR8 complex is shown in red color, whereas similar conformational complexes are shown in gray color. (**B**) The final selected bTLR8 complex superimposed with similar orientation complexes yielded by both docking programs. A chosen bTLR8 complex is shown in red color, whereas similar conformational complexes are shown in gray color. (**C**) The final selected pTLR8 complex superimposed with similar orientation complexes yielded by both docking programs. A chosen pTLR8 complex is shown in red color, whereas similar conformational complexes are shown in gray color. (**D**) The final selected mTLR8 complex superimposed with similar orientation complexes yielded by both docking programs. A chosen mTLR8 complex is shown in red color, whereas similar conformational complexes are shown in gray color. (**E**) The final selected rTLR8 complex superimposed with similar orientation complexes yielded by both docking programs. A chosen rTLR8 complex is shown in red color, whereas similar conformational complexes are shown in gray color.
(TIF)

Figure S4 **Dimer structure comparison between TLR3 and TLR8 dimers.** For comparison purposes, the available crystal dimer structure of TLR3 and dsRNA is shown in ribbon representation in brown and blue color, respectively, whereas the docked model structure of TLR8, synthetic small compounds ligands of R837 and R848 represented as ribbon and sticks are shown in (red and green) and blue, respectively.
(TIF)

Figure S5 **Ligand binding mode comparsion.** (**A**) Similar docked poses of R848 with hTLR8 are compared with each other, and the final selected docked pose is shown in red color stick representation. (**B**) Similar docked poses of R848 with bTLR8 are compared with each other, and the final selected docked pose is shown in red color stick representation. (**C**) Similar docked poses of R848 with pTLR8 are compared with each other, and the final selected docked pose is shown in red color stick representation. (**D**) Similar docked poses of R848 with mTLR8 are compared with each other, and the final selected docked pose is shown in red color stick representation. (**E**) Similar docked poses of R848 with rTLR8 are compared with each other, and the final selected docked pose is shown in red color stick representation.
(TIF)

Figure S6 **Sequence alignments of TLR8 from different species.** Sequences were used from human (h; Homo sapiens), mouse (m; Mus musculus), rat (r; Rattus norvegicus), pig (Bos taurus) and cow (Sus scrofa), elephant (lox; Loxodonta africana), cat (Felis catus), chimpanzee (chml; Pan troglodytes), horse (hor; Equus caballus), sheep (ovi; ovis aries), and water buffalo (wbu; Bubalus bubalis). The significant differences among the sepecies are highlighted in the black box.
(TIF)

Figure S7 **Phylogenetic tree of TLR8.** The evoultionary relationship calculated on the basis of the protein sequences of their TLR domains. human (h; *Homo sapiens*), mouse (m; *Mus musculus*), rat (r; *Rattus norvegicus*), pig (*Bos taurus*), cow (*Sus scrofa*), elephant (lox; *Loxodonta africana*), cat (*Felis catus*), chimpanzee (chml; *Pan troglodytes*), horse (hor; *Equus caballus*), sheep (ovi; *Ovis aries*), and water buffalo (wbu; *Bubalus bubalis*).
(TIF)

Table S1 **Ranking and interaction area of the selected docking models.**
(DOC)

Table S2 **The docking study of homodimer TLR8 complex contact residues among species.**
(DOC)

Table S3 **Interaction table of hTLR8/hTLR8-R847.**
(DOC)

Table S4 **Interaction table of bTLR8/bTLR8-R847.**
(DOC)

Table S5 **Interaction table of pTLR8/pTLR8-R847.**
(DOC)

Table S6 **Interaction table of mTLR8/mTLR8-R847.**
(DOC)

Table S7 **Interaction table of rTLR8/rTLR8-R847.**
(DOC)
