# Supplementary figures and images for: Comparative Analysis of Species-Specific Ligand Recognition in Toll-Like Receptor 8 Signaling: A Hypothesis
Source: PLoS One. 2011 Sep 20;6(9):e25118. doi: 10.1371/journal.pone.0025118 (PMC3176813; doi:10.1371/journal.pone.0025118)

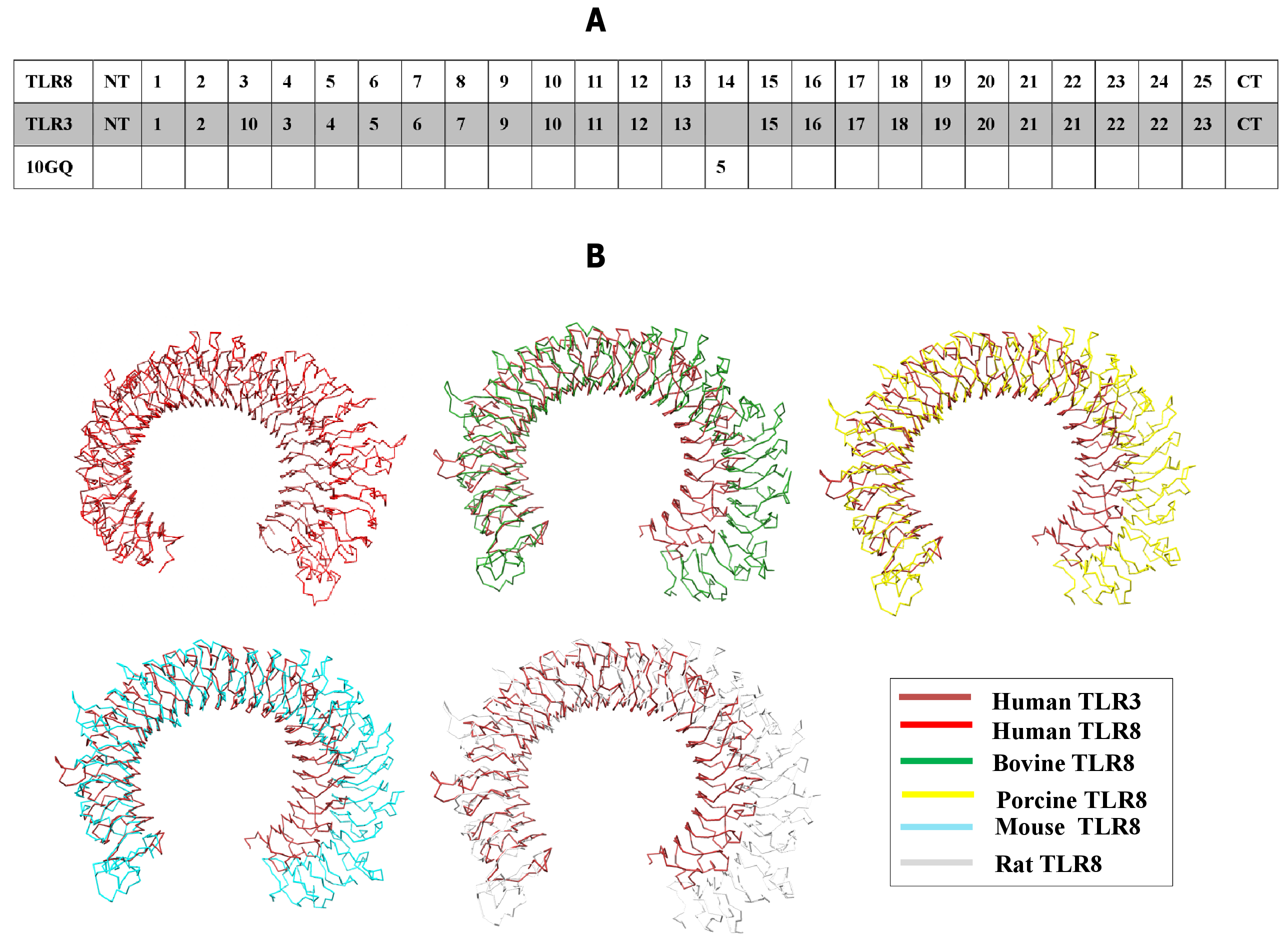

Supplement: Figure S1 — Multiple sequence and structural alignments between targate and templates. (A) Three templates, mTLR3 (3CIY), hTLR3 (1ZIW), and P. vulgarism polygalacturonase-inhibiting protein (1OGQ), were used to construct the model of the TLR8s. The number 1–25 indicates the canonical LRRs, and NT and CT indicate N- and C-terminal LRRs, respectively. (B) Superimposition between crystal TLR3 (red) and the MD-refined TLR8 models. (TIF) [file pone.0025118.s001.tif]

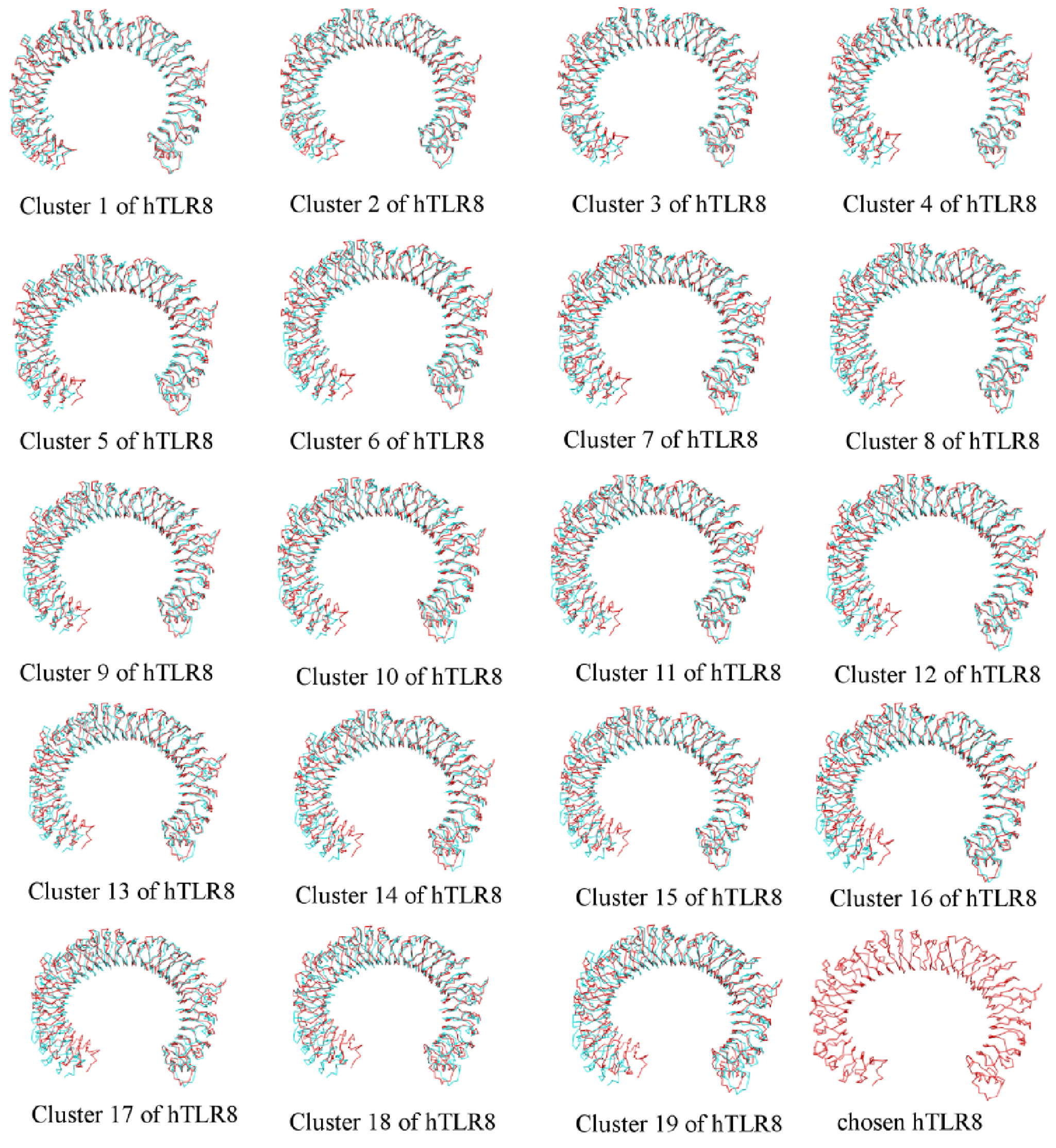

Supplement: Figure S2 — MDS-refined average structures of clusters. MDS produced 20 refined models of hTLR8 shown in alpha-carbon. Reference structure is cyan; the selected average structure of the cluster is red. Similar refined structures produced by MDS for the remaining TLR8 species were used for further studies. (TIF) [file pone.0025118.s002.tif]

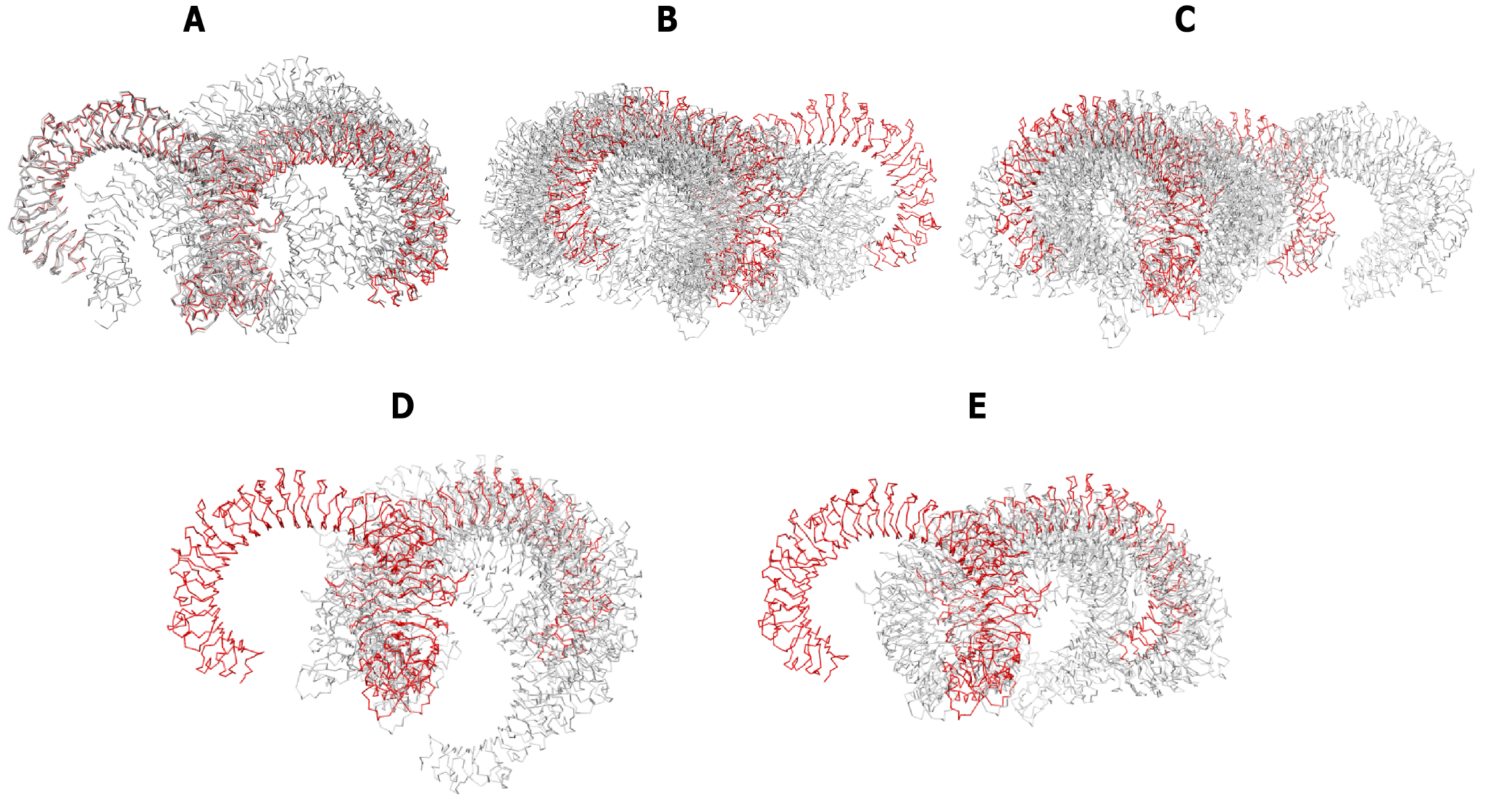

Supplement: Figure S3 — Top-ranked docked complex comparison with final selected complexes. (A) The final selected hTLR8 complex superimposed with similar orientation complexes yielded by both docking programs. A chosen hTLR8 complex is shown in red color, whereas similar conformational complexes are shown in gray color. (B) The final selected bTLR8 complex superimposed with similar orientation complexes yielded by both docking programs. A chosen bTLR8 complex is shown in red color, whereas similar conformational complexes are shown in gray color. (C) The final selected pTLR8 complex superimposed with similar orientation complexes yielded by both docking programs. A chosen pTLR8 complex is shown in red color, whereas similar conformational complexes are shown in gray color. (D) The final selected mTLR8 complex superimposed with similar orientation complexes yielded by both docking programs. A chosen mTLR8 complex is shown in red color, whereas similar conformational complexes are shown in gray color. (E) The final selected rTLR8 complex superimposed with similar orientation complexes yielded by both docking programs. A chosen rTLR8 complex is shown in red color, whereas similar conformational complexes are shown in gray color. (TIF) [file pone.0025118.s003.tif]

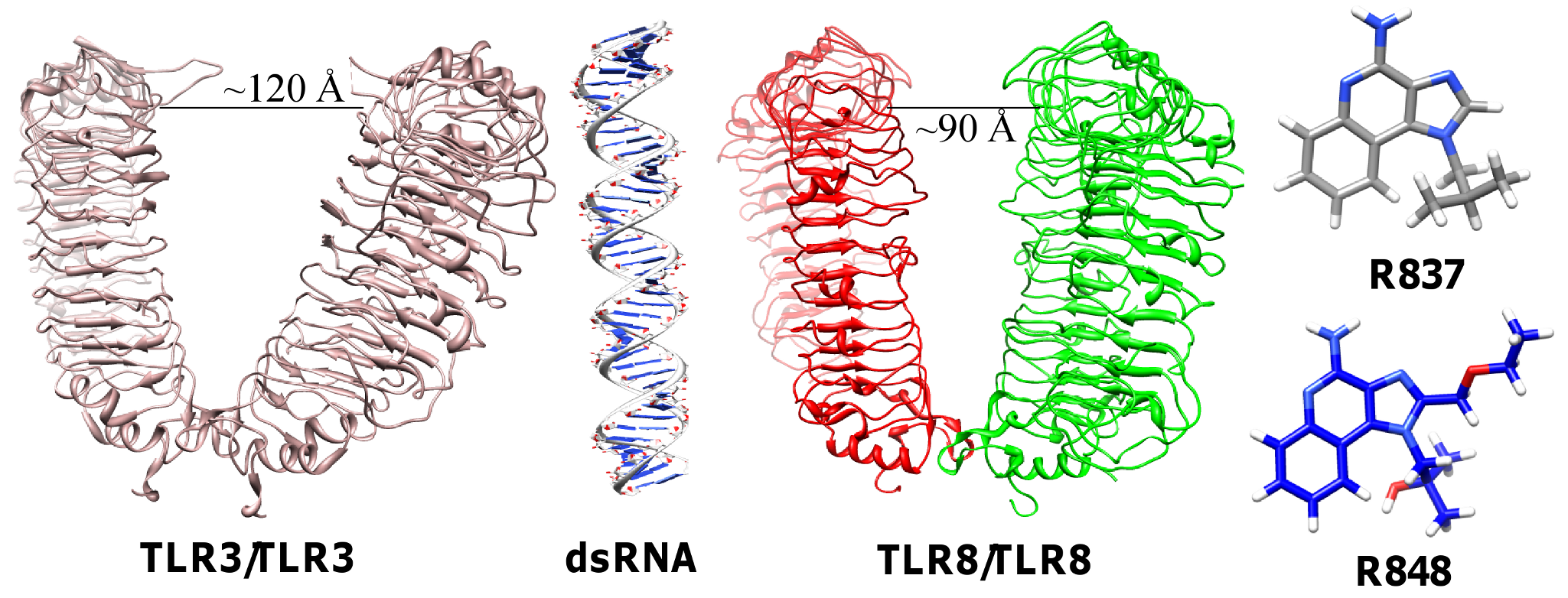

Supplement: Figure S4 — Dimer structure comparison between TLR3 and TLR8 dimers. For comparison purposes, the available crystal dimer structure of TLR3 and dsRNA is shown in ribbon representation in brown and blue color, respectively, whereas the docked model structure of TLR8, synthetic small compounds ligands of R837 and R848 represented as ribbon and sticks are shown in (red and green) and blue, respectively. (TIF) [file pone.0025118.s004.tif]

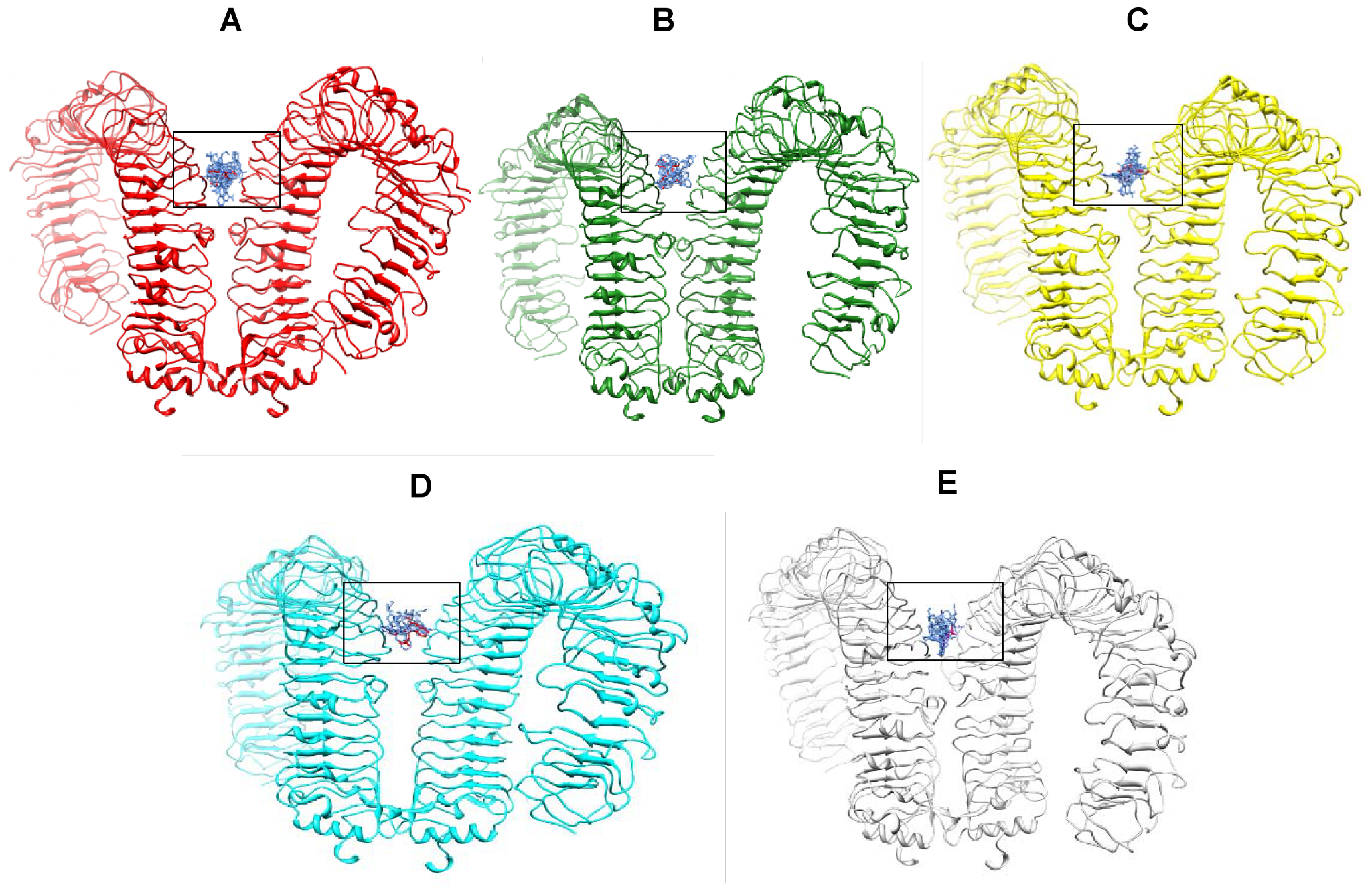

Supplement: Figure S5 — Ligand binding mode comparsion. (A) Similar docked poses of R848 with hTLR8 are compared with each other, and the final selected docked pose is shown in red color stick representation. (B) Similar docked poses of R848 with bTLR8 are compared with each other, and the final selected docked pose is shown in red color stick representation. (C) Similar docked poses of R848 with pTLR8 are compared with each other, and the final selected docked pose is shown in red color stick representation. (D) Similar docked poses of R848 with mTLR8 are compared with each other, and the final selected docked pose is shown in red color stick representation. (E) Similar docked poses of R848 with rTLR8 are compared with each other, and the final selected docked pose is shown in red color stick representation. (TIF) [file pone.0025118.s005.tif]

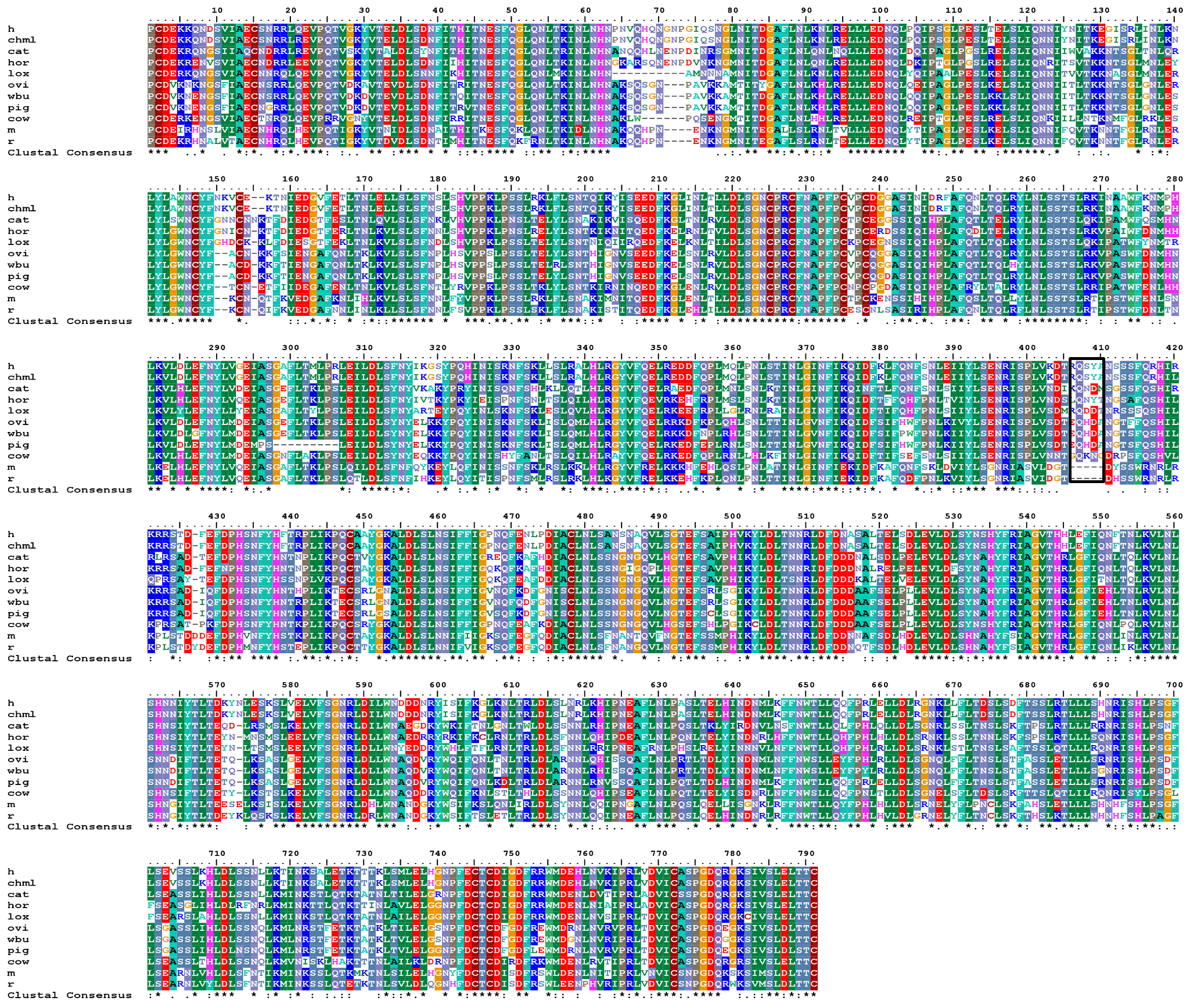

Supplement: Figure S6 — Sequence alignments of TLR8 from different species. Sequences were used from human (h; Homo sapiens), mouse (m; Mus musculus), rat (r; Rattus norvegicus), pig (Bos taurus) and cow (Sus scrofa), elephant (lox; Loxodonta africana), cat (Felis catus), chimpanzee (chml; Pan troglodytes), horse (hor; Equus caballus), sheep (ovi; ovis aries), and water buffalo (wbu; Bubalus bubalis). The significant differences among the sepecies are highlighted in the black box. (TIF) [file pone.0025118.s006.tif]

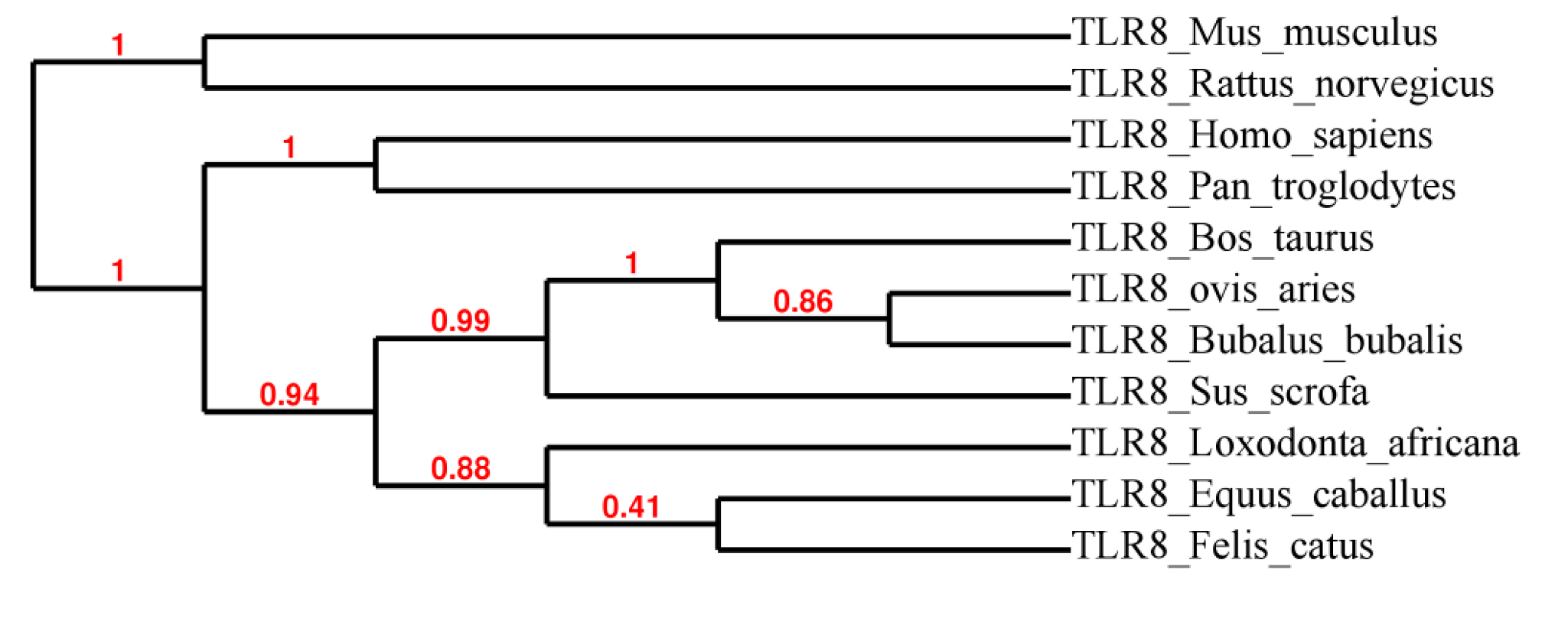

Supplement: Figure S7 — Phylogenetic tree of TLR8. The evoultionary relationship calculated on the basis of the protein sequences of their TLR domains. human (h; Homo sapiens), mouse (m; Mus musculus), rat (r; Rattus norvegicus), pig (Bos taurus), cow (Sus scrofa), elephant (lox; Loxodonta africana), cat (Felis catus), chimpanzee (chml; Pan troglodytes), horse (hor; Equus caballus), sheep (ovi; Ovis aries), and water buffalo (wbu; Bubalus bubalis). (TIF) [file pone.0025118.s007.tif]
