# Supplementary material for: Comparative Analysis of Species-Specific Ligand Recognition in Toll-Like Receptor 8 Signaling: A Hypothesis
Source: PLoS One. 2011 Sep 20;6(9):e25118. doi: 10.1371/journal.pone.0025118 (PMC3176813; doi:10.1371/journal.pone.0025118)
Supplement: Table S1 — Ranking and interaction area of the selected docking models. (DOC) [file pone.0025118.s008.doc]

**Table S1. Ranking and interaction area of the selected docking models.**

| **Complexes** | **GRAMM-X** | **Cluspro 2.0** | **Interaction Area(Å)** |
| --- | --- | --- | --- |
| hTLR8/hTLR8 | 3 | 7 | 706.8 |
| bTLR8/bTLR8 | 5 | 15 | 708 |
| pTLR8/pTLR8 | 6 | 10 | 721.7 |
| mTLR8/mTLR8 | 3 | 12 | 666.8 |
| rTLR8/rTLR8 | 2 | 7 | 698.5 |
