# Supplementary material for: Comparative Analysis of Species-Specific Ligand Recognition in Toll-Like Receptor 8 Signaling: A Hypothesis
Source: PLoS One. 2011 Sep 20;6(9):e25118. doi: 10.1371/journal.pone.0025118 (PMC3176813; doi:10.1371/journal.pone.0025118)
Supplement: Table S2 — The docking study of homodimer TLR8 complex contact residues among species. (DOC) [file pone.0025118.s009.doc]

**Table S2. The docking study of homodimer TLR8 complex contact residues among species**

| **Position** | **hTLR8** | **mTLR8** | **rTLR8** | **bTLR8** | **pTLR8** |
| --- | --- | --- | --- | --- | --- |
| 805 | N | N | ***S*** | ***S*** | ***S*** |
| 807 | G | G | G | G | G |
| 810 | R | ***K*** | R | ***E*** | R |
| 775 | E | ***D*** | ***D*** | ***D*** | ***D*** |
| 777 | T | T | T | T | T |
| 779 | D | D | D | D | D |
| 749 | K | K | K | K | K |

Note: The residues are not conserved among the species are shown in boldface with italic. hTLR8, mTLR8, rTLR8, bTLR8, and pTLR8 indicate human, mouse, rat, bovine, and porcine Toll-like receptor 8. Position represents the residue number in hTLR8.
