# Supplementary material for: Comparative Analysis of Species-Specific Ligand Recognition in Toll-Like Receptor 8 Signaling: A Hypothesis
Source: PLoS One. 2011 Sep 20;6(9):e25118. doi: 10.1371/journal.pone.0025118 (PMC3176813; doi:10.1371/journal.pone.0025118)
Supplement: Table S3 — Interaction table of hTLR8/hTLR8-R847. (DOC) [file pone.0025118.s010.doc]

**Table S3. Interaction table of hTLR8/hTLR8-R847**

| **Hydrogen bonds** | **Polar** | **Hydrophobic** | **Other** |
| --- | --- | --- | --- |
| **N2**- R541 (O) | **O1, H1**- H566 (ND1) | **C8**- L542 (CD2) | **C2, C7**- H566 (ND1) |
| **N3**- L542 (O) |  | **C2**- H566 (CE1) | **C8**-H566(ND1,NE2) |
| **N3**- D543 (O) |  | **C8**- H566 (CE1) | **O1**- H566 (CE1) |
| **N2**- Y567 (O) |  | **C3**- L543 (CD2) | **N1,N2**- L542 (CD2) |
| **N2**- F568 (O) |  | **C4, C5**- L542 (CD2) | **C9**- H566 (CD2) |
| **N4**- F568 (CB,CD1,CG,O) |  | **C1**- L542 (CD2) | **C16** –R569 (CB) |
| **H2, H3**- F568 (O) |  | **C9**- H566 (CE1) | **C17**- R569 (CB) |

Note: The residues from R848 that interact protein are shown in boldface.
