# Supplementary material for: Comparative Analysis of Species-Specific Ligand Recognition in Toll-Like Receptor 8 Signaling: A Hypothesis
Source: PLoS One. 2011 Sep 20;6(9):e25118. doi: 10.1371/journal.pone.0025118 (PMC3176813; doi:10.1371/journal.pone.0025118)
Supplement: Table S4 — Interaction table of bTLR8/bTLR8-R847. (DOC) [file pone.0025118.s011.doc]

**Table S4. Interaction table of bTLR8/bTLR8-R847**

| **Hydrogen bonds** | **pi-pi** | **Hydrophobic** | **Other** |
| --- | --- | --- | --- |
| **N2**- L526 (O) | **C3**- F528 (CB, CD1) | **C16**- L526 (CB) | **N1**- F528 (CB, C1) |
| **N4**- D527 (O) | **C4**- F528 (CB) | **C1**- F528 (CB) | **N2**- F528(CB) |
| **N4**- F552 (O) | **C5**- F528 (CB) | **C10**- F555 (CB) | **C13**- R553 (CD,CZ, NE,NH2) |
| **N3**- R553 (CG,O) | **C6**- F528(CD1) | **C13**- F555 (CB) | **C15**-R553 (CZ,NE,NH2) |
| **H3**- R553 (O) | **C12**- F528 (CD1) | **C15**- F555 (CB) |  |

Note: The residues from R848 that interact with protein are shown in boldface.
