# Supplementary material for: Comparative Analysis of Species-Specific Ligand Recognition in Toll-Like Receptor 8 Signaling: A Hypothesis
Source: PLoS One. 2011 Sep 20;6(9):e25118. doi: 10.1371/journal.pone.0025118 (PMC3176813; doi:10.1371/journal.pone.0025118)
Supplement: Table S5 — Interaction table of pTLR8/pTLR8-R847. (DOC) [file pone.0025118.s012.doc]

**Table S5. Interaction table of pTLR8/pTLR8-R847**

| **Hydrogen Bonds** | **Polar** | **Hydrophobic** | **pi-pi** | **other** |
| --- | --- | --- | --- | --- |
| **N2**-R531 (O) | **O2**-556(ND1) | **C13**-A561 (CB) | **C13**-F534 | **N2**-H556(CE1) |
| **N4**-R531 (O) | **O1**-H556 (ND1, NE2) | **C15**-A561(CB) | **C15**-F534 | **O2**-H556(CE1) |
| **N3**-D533 (O) |  | **C8**-L532(CG) |  | **O1**-H556(CD2, CE1, CG) |
| **N4**-D533 (O) |  | **C7**-H556(CD2, CE1) |  | **C2**-H556(NE2) |
| **N3**-F558 (O) |  | **C2**-H556(CE1) |  | **C7**-H556(NE2) |
| **N4**-F558 (CB,CD1,CE1,CG, O) |  | **C8**-H556(CE1) |  | **C8**-H556(NE2) |
| **H2**-D533 (O) |  |  |  |  |
| **H3**-N554 (O) |  |  |  |  |
| **H2**-F558 (O) |  |  |  |  |
| **H3**-F558 (O) |  |  |  |  |

Note: The residues from R848 that interact with protein are shown in boldface.
