# Supplementary material for: Comparative Analysis of Species-Specific Ligand Recognition in Toll-Like Receptor 8 Signaling: A Hypothesis
Source: PLoS One. 2011 Sep 20;6(9):e25118. doi: 10.1371/journal.pone.0025118 (PMC3176813; doi:10.1371/journal.pone.0025118)
Supplement: Table S6 — Interaction table of mTLR8/mTLR8-R847. (DOC) [file pone.0025118.s013.doc]

**Table S6. Interaction table of mTLR8/mTLR8-R847**

| **Hydrogen Bonds** | **Hydrophobic** | **pi-pi** | **Other** |
| --- | --- | --- | --- |
| **O1**-H557(ND1) | **C3**-L533 (CD2) | **C12**-H557(CE1) | **N3**-L533(CD2) |
| **H1**-H557(ND1) | **C5**-L533(CD2) | **C14**-H557(CE1) | **C12**-H557(ND1) |
| **O2**-H557(ND1) | **C6**-L533(CD2) | **C15**-F559(CD1) | **C14**-H557(ND1) |
|  | **C10**-L533(CD2) |  | **C1**-H557(ND1) |
|  | **C1**-H557(CB,CG) |  | **C7**-H557 |
|  | **C7**-H557(CB) |  | **H3**-L533(CD1,CD2,CG) |
|  | **C8**-H557(CB,CG) |  | **N2,N4,H2**-L533(CD2) |
|  |  |  | **C8, C16**-H557(ND1) |
|  |  |  | **O2**-H557(CE1) |

Note: The residues from R848 that interact with protein are shown in boldface.
