# Supplementary material for: Comparative Analysis of Species-Specific Ligand Recognition in Toll-Like Receptor 8 Signaling: A Hypothesis
Source: PLoS One. 2011 Sep 20;6(9):e25118. doi: 10.1371/journal.pone.0025118 (PMC3176813; doi:10.1371/journal.pone.0025118)
Supplement: Table S7 — Interaction table of rTLR8/rTLR8-R847. (DOC) [file pone.0025118.s014.doc]

**Table S7. Interaction table of rTLR8/rTLR8-R847**

| **Hydrogen Bonds** | **Hydrophobic** | **pi-pi** | **Other** |
| --- | --- | --- | --- |
| **N4**-H544(CD2,CE1,NE2) | **C8**-F532(CD,CG,NE) | **C13**-H554(CE1) | H1-L530(CB.CD1,CG) |
| **H2**-R529(NE2) | **C1**-F532(CD1) | **C15**-H554(CE1) | **O1**-L530(CD1) |
| **H3**-R529(NE2) | **C16**-F482(CE2) |  | **C13,C15**-H554(ND1) |
| **H3**-L530(NE2) | **C17**-F482(CE2) |  | **C5.C11**-R529(NE) |
| **H2**-H554(NE2) | **C11**-L530(CD2) |  | **C17**-R529(CD,NE) |
| **H3**-H554(NE2) | **C17**-L530(CD2) |  | **C4,C9**-R529(NH2) |
|  |  |  | **N4**-L530(CD2) |

Note: The residues from R848 that interact with protein are shown in boldface.
